# Resource Allocation Schemes for 5G Network: A Systematic Review

**DOI:** 10.3390/s21196588

**Published:** 2021-10-02

**Authors:** Muhammad Ayoub Kamal, Hafiz Wahab Raza, Muhammad Mansoor Alam, Mazliham Mohd Su’ud, Aznida binti Abu Bakar Sajak

**Affiliations:** 1 Malaysian Institute of Information Technology (MIIT), Universiti Kuala Lumpur, Kuala Lumpur 50250, Malaysia or ayoub.kamal@iobm.edu.pk (M.A.K.); raza.hafiz@s.unikl.edu.my (H.W.R.); or m.mansoor@riphah.edu.pk (M.M.A.); 2 Institute of Business and Management, Karachi 75190, Pakistan; 3 Riphah Institute of System Engineering (RISE), Faculty of Computing, Riphah International University, Islamabad 46000, Pakistan; 4 Malaysian France Institute (MFI), Universiti Kuala Lumpur, Kuala Lumpur 50250, Malaysia; mazliaham@unikl.edu.my

**Keywords:** 5G, resource allocation, resource distribution, congestion, 5G communication, comprehensive, review, systematic

## Abstract

Fifth-generation (5G) communication technology is intended to offer higher data rates, outstanding user exposure, lower power consumption, and extremely short latency. Such cellular networks will implement a diverse multi-layer model comprising device-to-device networks, macro-cells, and different categories of small cells to assist customers with desired quality-of-service (QoS). This multi-layer model affects several studies that confront utilizing interference management and resource allocation in 5G networks. With the growing need for cellular service and the limited resources to provide it, capably handling network traffic and operation has become a problem of resource distribution. One of the utmost serious problems is to alleviate the jamming in the network in support of having a better QoS. However, although a limited number of review papers have been written on resource distribution, no review papers have been written specifically on 5G resource allocation. Hence, this article analyzes the issue of resource allocation by classifying the various resource allocation schemes in 5G that have been reported in the literature and assessing their ability to enhance service quality. This survey bases its discussion on the metrics that are used to evaluate network performance. After consideration of the current evidence on resource allocation methods in 5G, the review hopes to empower scholars by suggesting future research areas on which to focus.

## 1. Introduction

The remarkable progress in data communication has had a radical influence on wireless networks. Predictably, the quantity of wireless devices has continued to rise at an enormous rate [1]. Shortly, an even more mobile and connected society will emerge, defined by massive increases in connection, traffic volume, and a far larger range of usage scenarios. The amount of traffic will increase dramatically. Between 2010 and 2030, worldwide data traffic is expected to rise by more than 20,000 times. Though smart phones are anticipated to tend to be the most popular personal devices, the number of other types of devices, such as wearables and smart devices, is expected to rise. Consequently, the fifth-generation (5G) cellular communications system should be broadly introduced to satisfy the continuously evolving demands that prior generations of systems were unable to meet [2].

Despite the advancements in 4G wireless network technology, providing mobile services that demand high speed, fast response, high dependability, and energy efficiency is difficult. As a result, these functionalities have become critical needs for future 5G services. Current 4G/LTE networks are incapable of providing immediate cloud services, interactive Internet, enhanced vehicle-to-everything (eV2X), Internet of Things (IoT), and connectivity with drones and robotics, all while maintaining a high level of user experience [3]. As a result, the world has seen plenty of technical improvements in the domain of transmission. Currently, mobiles have everything, varying from the smallest size, video, and audio call support to enormous phone processors [4] and memory that contends with the modern laptops in the marketplace [5].

This innovative trend in technological transformation is altering the methods by which we live, work, and interconnect with everyone [6]. We have realized the emergence of extraordinary services and applications—for example, autonomous vehicles, artificial intelligence [7], smart homes, smart factories, smart cities, and drone-based delivery systems, etc. The collaboration between apparatus and human-based assistance will expand the forthcoming wireless environments with cost effectiveness challenges [8]. Forthcoming increases in cell phone communication capabilities will saturate all aspects of public life and will generate a multidimensional, consumer-related ecosystem.

Furthermore, an entire mobile-based linked environment is anticipated, characterized by a greater amount of traffic, a much wider span of running consequences, and an amazing volume of expansion in connectivity [9]. This extraordinary heightening of traffic suggests that mobile networks will have to deliver approximately a thousand times the spectral effectiveness of the current decade’s existing structure [10]. Furthermore, a spectrum efficiency (SE) enhancement of 5 ≅ 15 times was related to mobile networks of the fourth generation (4G) [11].

The 5G network incorporates numerous technologies—for example, Internet of Things (IoT) [12,13], software-defined networking (SDN) [14], device-to-device (D2D) communications [15], vehicular networking [16], machine-to-machine (M2M) communications [17], unmanned aerial vehicles (UAV) [18], cloud radio access networks (CRANs) [19], mobile edge computing (MEC) [20], and cloud computing [21]—to allow the traditional communication network to realize an Internet of everything [22]. Preserving the tempo of progress towards meeting this extreme need will demand that leading-edge technologies increase the enormous cellular capability that is envisioned in the acclaimed 5G cellular structures.

Significant academic and industry-based research studies have been conducted to overcome the abovementioned challenges, and they have stressed the importance of wireless structures that offer improved spectral proficiency and broader bandwidth than the present cellular networks via the placement of several antenna components and frequency reuse [23,24]. The IoT is a dominating force—even at this present moment in time—with its enormous number of wireless apparatuses, such as sensors, smartphones, tablets, and machines. To transfer enormous amounts of data, which can traffic at speeds varying up to 100 Gbps/km^2^ through elevated enhanced mobility, such machines require supplementary well-organized and pervasive radio access technologies (RATs) [25].

Concerning the challenge of the anticipated explosive increase in the amount of traffic, the radio obstruction and resource management techniques of RAN in 5G systems will have to accommodate more than 1000× the existing traffic volume. Furthermore, the data comprising the enormous full extent of this traffic will have to be accessible and distributable anytime, anywhere, and by anything or anyone inside the 5G RAN and outside the 4G cellular pattern [26].

Hence, mobile network operators (MNOs) are projected to encounter tough environments to elevate the performance of the network. Furthermore, cutting-edge applications have various service prerequisites with regard to energy consumption and latency [27]. For the past decade, scholars in the domain have been mainly concerned with pioneering state-of-the-art solutions, along with messy ideas and technologies, all to stay steps or even leaps ahead of the existing cellular systems and their identified drawbacks [11]. IoT is projected to empower an environment that will enhance numerous aspects of normal everyday life, as well as providing professional applications that will play a role in increasing the world economy once it achieves the critical mass that comes from being applied to a wide variety applications [28].

Large-scale applications of IoT require a huge configuration of linked smart machines that might be installed in such a wide variety of areas as agricultural monitoring, shipping environments, smart health systems, smart cities, smart homes, etc. [29], all of which require common access to the cloud, resulting in substantial cost efficiency. For example, visualize a situation in smart homes, where people will be capable of employing this technology without human intervention for opening a garage door when coming home, turning on the lights or a particular set of them, regulating the heating/cooling system, turning on the coffeemaker to make early morning coffee, and many other smart applications for various purposes [30].

Ref. [31] asserts that as the range devices continues to expand enormously, along with the service categories, a user’s or client’s demand for excellence also increases. The ever-growing volume of network data traffic has become a serious problem. Hence, network traffic handling, mostly in the future 5G cellular dissimilar networks [32,33] and ultra-dense networks (UDNs) [34,35], is likely to be a precarious problem due to the important pressure imposed on wireless communication networks by the traffic caused by the rising volume of large amounts of data.

**Figure 1:**
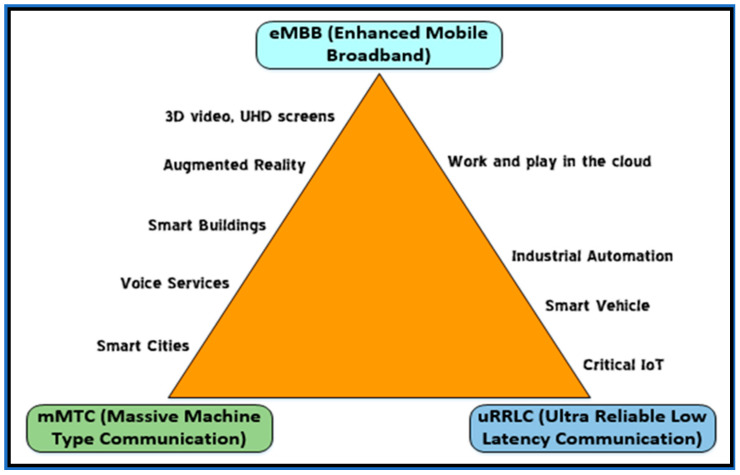
Usage scenario of IMT for 5G.

The 5G (CNs) have a cellular means for the provision of satisfactory broadband wireless communication [35,36]. In the International Telecommunication Union (ITU), the 5G ITU-radiocommunication (ITU-R) operating class serves a function in the growth of 5G under International Mobile Telecommunication (IMT) 2020 [37]. As shown in the Figure 1 the vision of this effort is to accomplish 1000× throughput enhancement and 100 billion associations and to reduce latency near to 0 [35,38]. Certainly, 5G will improve enhanced mobile broadband (eMBB) through extended 100 Mbps data rates by the consistent spatial sharing of max bandwidth ranging from 10 to 20 Gbps [35,36].

Additionally, 5G will offer mobile facility, massive machine-type communications (mMTC), and dangerous latency facilities. In ultrareliable low latency communication (uRLLC), problems in reliability and latency requirements require attention [39]. In several situations, a consequent end-to-end (E2E) latency as small as 1 ms tends to happen with a consistency distinguished as 99.99% [40].

**Figure 2:**
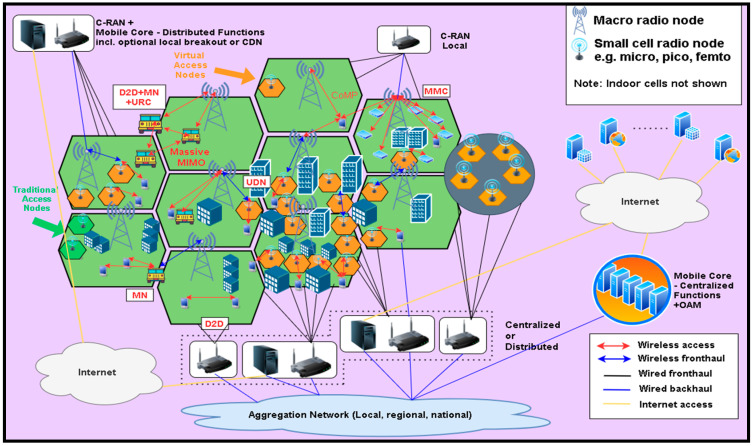
Generic 5G network design (high-level topological view) [41].

In Figure 2, a topological view of a generic 5G network is presented.

## 2. Background

**Figure 3:**
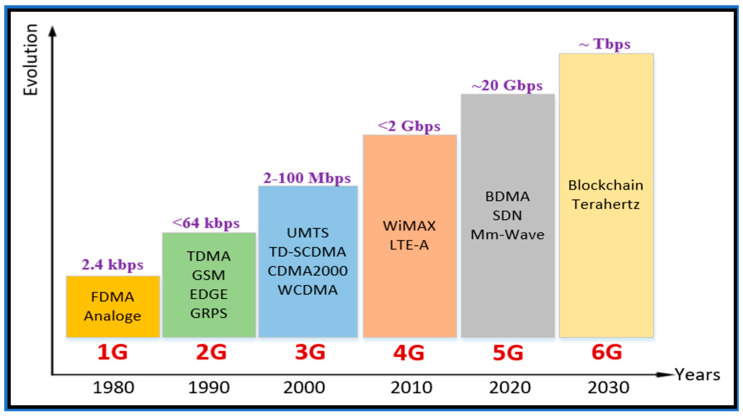
Data rate by technology: 1G to 6G [53].

As the demand for wireless technologies is increasing day by day, the coverage, data rate, spectral efficiency, and mobility are also frequently rising [42]. The improvements similarly demonstrate that the 1G and 2G technologies utilized circuit switching [43,44], while 2.5G and 3G utilized packet and circuit switching, respectively, as did the succeeding generations after 3.5G to present, i.e., [45], whereas 5G is suited to using packet switching. In addition to these aspects, it correspondingly distinguishes between the unlicensed and licensed spectrum. All developing generations used the accredited range, whereas Bluetooth, WiMAX, and Wi-Fi are utilizing the unlicensed range. An outline regarding the growing wireless technologies is discussed below and shown in Figure 3: The 1G was named in the early 1980s; it consisted of a max data rate of 2.4 kbps [46].

The main contributors were Total Access Communication System (TACS), Advanced Mobile Phone System (AMPS), and Nordic Mobile Telephone (NMT). The technology had various drawbacks corresponding to reckless handoff and below par capacity, with no security and inferior voice associations; meanwhile, voice calls remained held and playable in wireless towers, resulting in the susceptibility of these calls to uninvited snooping during or after the calls, due to the growth of third-party providers [47,48]. The 2G was announced in the 1990s by utilizing digital equipment in 2G cellular phones. The leading ability of 2G was the intention to introduce Global Systems for Mobile communications (GSM) that primarily utilized voice-over communication, with a capable data rate of 64 Kbps. In addition, the 2G mobile phone battery life was extended, although the wireless signals had little power. These services mentioned above offered capabilities similar to both electronic mail and Short Message Service (SMS). Energetic well-known technologies were GSM, Code Division Multiple Access (CDMA), and Interim Standard (IS) 95 [49,50].

Services normally subscribe to a 2G CN combined with General Packet Radio Services (GPRS), and extra capabilities are not frequently provided in 1G or 2G CN. A 2.5G CN mostly uses 2G system structures; nevertheless, it pertains to circuit switching in addition to packet switching. It facilitates a capable data rate of 144 kbps. The 2.5G key technologies remained CDMA 2000, Enhanced Data Rate for GSM Evolution (EDGE), and GPRS [51,52]. The 3G was launched in late 2000. It conveys a communication rate capable of 2 Mbps. The 3G structures combine extreme rate mobile access with services initiated on Internet Protocol (IP). Apart from the improved communication rate, progressive enhancement was prepared for maintaining the quality of service (QoS). Further services such as worldwide roaming and enhanced voice property led to 3G being billed as an extraordinary generation.

The key weakness of 3G phones is the requirement for some extra energy, as compared with the majority of 2G brands. In addition to this, 3G system strategies are more costly than 2G [51,52]. Meanwhile, 3G contains the operations of Universal Mobile Telecommunications Systems (UMTS) Wideband CDMA, Evolution-Data Optimized (EVDO), High-Speed Downlink Packet Access/High-Speed Uplink Packet Access (HSDPA/HSUPA), and CDMA 2000 equipment, which have built a central wireless organization amongst 3G and 4G called 3.5G, which employs a better-quality data rate of 5 to 30 Mbps [51]. Long-Term Evolution (LTE) and Static Worldwide Interoperability for Microwave Access (WiMAX) 3.75G are the upcoming mobile data amenities. Both Static WIMAX and LTE can increase the ability of the system and deliver a comprehensive variety of high-speed capabilities such as peer-to-peer file distribution, merged web facilities, and on-request video to a considerable quantity of customers having the ability of entree.

Moreover herewith, the associated range is able to recognize the operators to implement their system and to highlight the improved exposure, having better quality with minimum cost [49,52]. The 4G network is normally discussed as the successor of the 2G and 3G levels. The 3rd Generation Partnership Project (3GPP) is regulating LTE Advanced as a 4G level in addition to WiMAX. A 4G structure advances the existing transmission systems by conveying a whole and consistent answer built on IP. Capabilities such as data, multimedia, and voice will be communicated to subscribers on each stage, and all systems will contain much more bandwidth than previous creations. Appliances having a built-in 4G network include high-definition TV content, video chat, digital video broadcasting (DVB), mobile TV, and multimedia messaging service (MMS) [50].

Through a tremendous growth in the need of the consumers, 4G would be upgraded to 5G through an innovative technology called Beam Division Multiple Access (BDMA), Filter Bank Multicarrier (FBMC), or non- and quasi-orthogonal space–time block code [54]. The idea behind the BDMA method is described because of the situation having interaction among mobile stations and base stations. Here, in the transmission, an independent beam is associated with every mobile station. The method of BDMA splits that probe beam corresponding to the positions of the cellular stations aimed at providing numerous entrees to the cellular stations, which correspondingly increases the size of the structure [55]. In the future, to address the method’s assumptions and challenges, the recently established wireless networks will have to improve in several aspects. The current technological components of long-term evolution (LTE) and high-speed packet access (HSPA) are presented as a way to enhance the existing wireless technologies.

**Figure 4:**
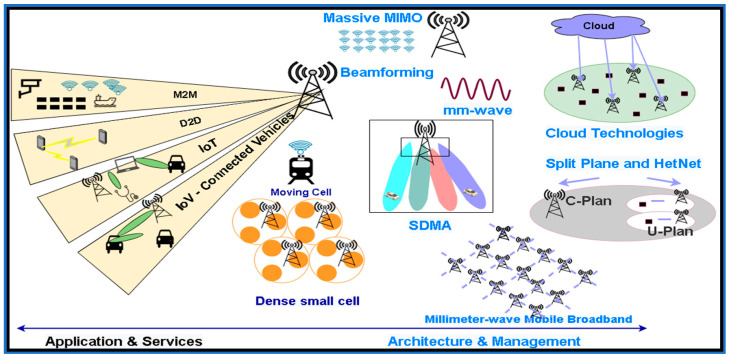
5G design and applications [35].

However, supporting devices may lead to the creation of upcoming novel wireless-based technologies, which might assist in the growth of future technologies. The technology behind these innovative apparatuses may have distinct approaches to retrieving spectrum and significantly advanced frequency limits, the beginning of enormous antenna organizations, ultra-dense locations, and direct device-to-device interaction [56]. The 5G system is expected to support the traffic of a huge amount of data and an enormous range of wireless connectivity [57], as shown in Figure 4. Dissimilar data traffic has distinct QoS prerequisites. The 5G mobile system aims to tackle the limits of preceding standards, which are a potentially important enabler for upcoming IoT.

The 5G systems promise to improve a broad span of applications— for example, multimedia and entertainment, Industrial IoT (IIoT), mission-critical applications, smart health, drone operations, autonomous driving, and smart home [58]. Nowadays Industry 4.0 is improving due to the upcoming rise and evolution of technologies such as biotechnology, quantum computing, and artificial intelligence, etc. [59].

This innovative evolutionary trend has changed our working style and living environment to one in which we relate to everyone and everywhere. As a result, we might realize the development of extraordinary support to such applications as smart factories, drone-based delivery systems, autonomous vehicles, smart homes, and artificial intelligence, etc. The presence of both apparatus and human-centric facilities would be distinguished by the upcoming wireless ecosystems [60]. Soon, communication using cellular connectivity will penetrate all segments of public life and will create a multidimensional, customer-centric information atmosphere. Furthermore, a completely mobile and connected society is expected to have an incredible and growing amount of traffic and connectivity and a far wider variety circumstances regarding data handling [9].

## 3. Related Work

The 5G network empowers connectivity amongst a huge number of apparatuses. This incredible increase in the number of apparatuses needs a broad spectrum of resources to support the function of any kind of application as well to deal with the enormous load the applications place on the BS. The best allocation of resources—for example, spectrum, time, and power—may increase the functioning of the system. This survey discusses and compares the existing resource allocation methods in 5G.

The authors of [1] included a systematic optimization taxonomy of different elements of resource allocation as well as a complete assessment of resource allocation strategies in a CRAN. They identified and explained the main aspects of effective resource allocation and management in CRAN, including throughput maximization, user assignment, spectrum management, remote radio heads (RRH) selection, power allocation, and network utility. In addition, the authors described new use-cases such as virtualized CRAN, heterogeneous CRAN, Orthogonal Multiple Access (NOMA)-based CRAN, millimeter-wave CRAN, and non- and full-duplex-enabled CRAN to show how CRAN technology may improve a system’s performance.

In [61], the authors outlined the challenges that may arise as a result of future 5G systems and emphasized their relevance. A survey technique was described, as well as the various methodologies utilized in recently published surveys classifying radio resource management (RRM) schemes. They reviewed the newly researched HetNet RRM methods, with an emphasis on the optimization of radio resource allocation in conjunction with other methods. These RRM schemes were divided into categories based on their optimization metrics, after which they were examined and contrasted qualitatively. The authors observed the complexity of RRM schemes in terms of implementation and computation.

Researchers in [62] presented a complete assessment on resource allocation (RA) in heterogeneous networks for 5G communications. First, they went through a description of HetNet and the various network situations. Second, the topic of RA models was explored. The authors next provided a categorization scheme for assessing current RA systems in the literature. Finally, several difficult outstanding questions and prospective research directions on the subject were discussed. The authors also presented two viable techniques for sixth-generation (6G) communications to tackle the RA issues of future HetNets—namely, a control theory-based approach and a learning-based approach.

The authors of [63] assessed the current state of such technological advancements. Relevant radio interference and resource management (RIRM) methods were the subject of attention. The authors’ contribution is based on their analysis, synthesis, and summary alignments of traditional RIRM methods in order to address the stated difficulties faced by 5G RAN systems. The paper identified a number of open research questions that have arisen as a result of newly suggested RIRM systems.

The authors of [64] focused on resource allocation algorithms in 5G network slicing, including its concepts and models. Initially, the essential concepts of software-defined networks (SDN) and network function virtualization (NFV), as well as their roles in network slicing, were introduced. Network slicing management and orchestration (MO) architecture, which offers a foundation for resource allocation algorithms, was also described. Then, in RAN slicing and core network (CN) slicing, resource categories with appropriate isolation levels were investigated. Furthermore, mathematical models of resource allocation algorithms were classified according to their goals, and they were illustrated with real-world examples. Additionally, viable solutions to open research challenges were identified in this study.

The study by the authors of [65] provided a thorough examination of resource allocation strategies for the two most common vehicular network technologies—namely Dedicated Short Range Communications (DSRC) and cellular-based vehicular networks. The authors explored resource allocation difficulties and possibilities in current vehicle networks, as well as a number of potential future research topics. The authors of [66] investigated resource management in 5G, covering the core network and RAN, and they classified current studies based on network architecture, application scenarios, and research aims. According to the authors’ conclusions, the studies that were classified faced several obstacles relevant to future research. The authors also shared possible future research ideas with readers in the hopes of encouraging other academics to explore issues related to 5G resource allocation.

The abovementioned studies focused on various domains of 5G with respect to resource allocation, such as RAN, C-RAN, HC-RAN, and CN, while this study focuses on the entire 5G network with respect to resource allocation. There was a need for a systematic literature review regarding resource allocation methods in 5G networks. Our study addresses this unmet need by conducting a systematic review and analysis of 5G resource allocation methods. In this study, five questions were formulated to unambiguously demonstrate the significance of resource allocation for 5G, keeping an eye on improving its consideration in future perspectives.

## 4. Design of Research

This part of the study focuses on the structure used to perform this systematic literature review, which is based on instructions for performing an SLR guided by [67], with specific emphasis on 5G resource allocation. The formation of research questions is the main part of an SLR, along with the factors of motivations that are presented in this portion. The included articles were chosen from multiple data sources. Specifically, a research strategy was created to concentrate on articles related to a specific domain, which is mentioned in this section. Subsequently, the research papers were collected for study based on evaluation criteria for inclusion and exclusion. Motivations and research questions were formulated to critically identify the state of the art of resource allocation in 5G.

### 4.1. Research Questions

The following are the focused research questions that were discussed and analyzed in this study:

1. What are the existing state-of-the-art challenges in 5G?
2. What is the importance of resource allocation in 5G?
3. Which current policies, strategies, and algorithms are being used for resource allocation in 5G?
4. Which metrics and parameters are considered during resource allocation in 5G?
5. Which open issues and research trends are unaddressed in resource allocation in 5G?

### 4.2. Search Criteria

A systematic resource allocation analysis was completed using well-known research. The main emphasis was on 5G, as this is more connected to IoT and improved monitoring and network performance. Because there was no current research performed in the area, the articles taken for consideration in this SLR were from 2015 onward. Depending on the research questions and the proposed theme, we present the search terms that were used for seeding purposes to identify an initial set of articles for consideration. The research team entered terms for searching, including “5G communication”, “resource allocation”, which were nominated for main keywords. We applied the “OR” and “AND” logical operators for connecting the important search terms. Later, after performing limited tests, we selected the associated search string that provided us with sufficient relevant research articles by utilizing the keywords to frame the search strings presented below in Table 1.

### 4.3. Data Sources

For this SLR, many diverse data sources were investigated. The databases in Google Scholar, Scopus, were examined mostly for conference reports, journal papers, magazines, and books relevant for inclusion. In addition, publishers of high-quality articles such as Springer, IEEE, Wiley, Science Direct, Sage, Google Scholar, MDPI, ACM digital library, etc. included for review, as shown below in Table 2.

### 4.4. Article Selection Process

**Figure 5:**
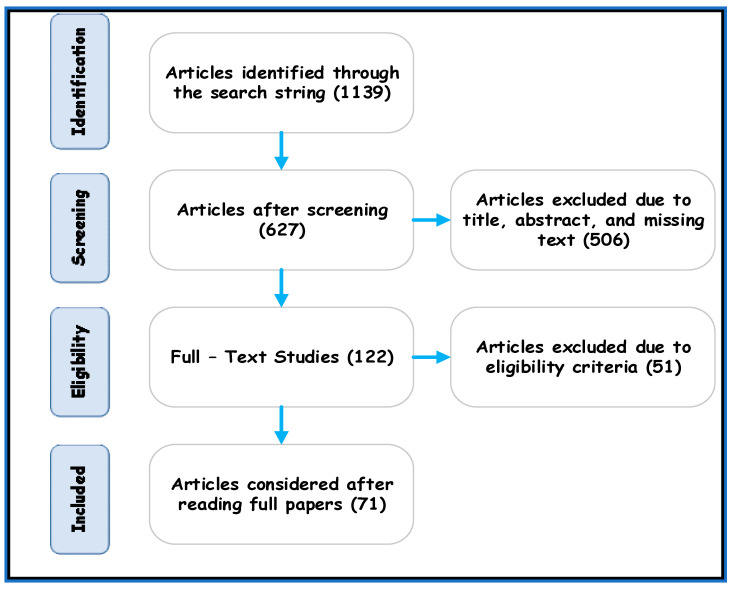
Article selection procedure [68,69].

The perspective of a research article was the predominant way through which several quantitative kinds of research were selected. Quality assessment principles were applied to certain articles to determine their exclusion and inclusion. The tactic used to select the articles started with formulating the research questions, as mentioned above. Outlining the string of searches supported the search and selection procedure. Only English language articles were studied in this review. The PRISMA flow diagram [68,69] was followed and is shown in Figure 5. After obtaining the initial research articles based on the strings and keywords, we reviewed how resource allocation schemes in 5G communication were addressed in each article. The search procedure finished by classifying the resource allocation scheme to ensure the comprehensiveness of this study. Several papers were eliminated due to a mismatch between their titles and the strength of the measures that were used. Additionally, abstracts on their own were not considered for inclusion in this study.

As presented in Figure 5, a total of 1139 research articles were collected using the search strings, as a result of the aforementioned inquiry. The articles were published from the year 2015 to 2020 in various quality journals and other publications, as presented in Table 2. To select compelling research articles, the criteria of inclusion and exclusion were applied, as shown in Table 3, to reduce their number to 627. Based on the abstracts and titles, the selection was reduced to 122 articles related to the selected domain. From this point onward, these 122 articles were examined, and they were categorized bases on their resource allocation techniques as conventional or artificially intelligent in 5G communication: 71 articles were ultimately selected.

Depending on the selection criteria, the most relevant articles based on abstracts, title, and comprehensive research were selected, ensuring that the results would be relevant to the research work as desired.

### 4.5. Inclusion and Exclusion Criteria

**Figure 6:**
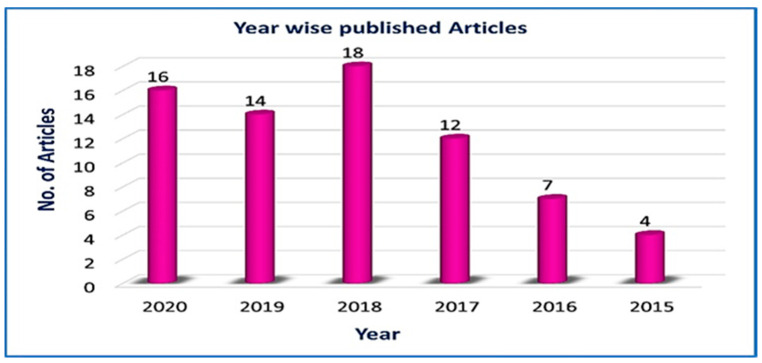
Selection of articles for review by year of publication.

The articles chosen for this study are shown in Figure 6, which presents by year of publication the articles selected for this review. The selection of these articles was further classified by publisher and by the methods used for resource allocation in 5G.

In the end, there were 71 articles considered for this study of 5G resource allocation. These articles were taken from various well-known research journals, such as IEEE, Springer, Elsevier, Wiley, MDPI, ACM, and some other publishers.

**Figure 7:**
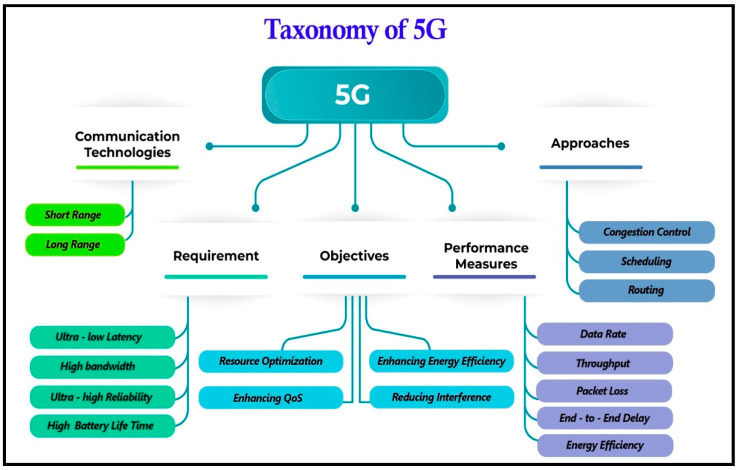
Taxonomy of 5G.

As shown in Figure 7 the parameters that make up the stated 5G taxonomy are, 1- Requirements, 2- Objectives, 3- Performance metrics, and 4- Approaches 5- Communication technologies.

## 5. Discussion

The literature review uncovered several findings across each research question, as discussed below:

### 5.1. Q1. What Are the Existing State-of-the-Art Challenges in 5G?

The following challenges in 5G communication came to be known after studying several research papers.

The 5G communication challenges are:

- Deployment of MIMO: 5G will require a paradigm shift that incorporates a huge bandwidth having very high-frequency spectra as a carrier, excessive densities of a base station, and a remarkable number of antennas to provide for the massive growth on the behalf of the increased amount of traffic.
- mm-Wave: Millimeter waves are transmitted with frequencies between 30 and 300 GHz, compared with the bands traditionally used for mobile devices, which are below 6GHz. This technology guarantees huge data capacity as compared with the one that is currently being used. However, mm-waves face one main drawback—i.e., traditionally, a higher range of frequencies is not sufficient for outdoor applications due to blockage and high propagation loss caused by rain and tall buildings [70].
- Pilot contamination and channel estimation/feedback: Channel State Information (CSI) is critical for attaining the benefits of multi-antenna in MIMO systems. CSI has become more demanding in massive MIMO systems because of the massive number of antennas. Furthermore, a massive MIMO system needs a massive number of pilots for both times-division duplexing (TDD) and frequency-division duplexing (FDD) [71].
- The trade-off between computation power and transmission power: Across the 5G network, an additional BS’s power relies on the transmission and computation power of the BSs. When extra power is added to BSs and combined with the transmission and computation power of the additional BS, then the 5G network’s energy efficiency is also calculated by the BSs’ transmission and computation energy [72].
- Mobility: 5G networks require work with speed up to 1000 km/h [73,74]. A substantial investigation is needed to uncover the issues related to the selection of optimum beam and the development of methods/schemes that enhance the requirement for the response for CSI to the transmitter. Thus, massive MIMO performance is delicate with regard to speed because this the computational load can make multiuser solutions unaffordable [41].
- Mixed-Numerology interference: As per the divergent demands of mMTC and URLLC, service configuration contrasts vigorously from the perspectives of the physical layer [75]. Specifically, mMTC is characterized by a sampling rate having a low baseband for supporting huge connectivity and by a small sub-carrier spacing with narrowband transmission, reduced consumption of power, and extensive coverage having low-cost. On the contrary, URLLC mostly requires spacing for many subcarriers to deal with the rigorous requirement for latency and sampling rate for high baseband. These diverse configuration discrepancies in RF and baseband predictably lead to considerable interference [76,77] in crucial mMTC.
- 5G UE’s testing challenges: The problems that appeared in testing 5G UE are like the issues that happened in traditional systems having power control, extreme power output, and sensitivity behavior of receiver as measurement matrices. Therefore, the demand for using SC-FDMA for the uplink and an OFDMA scheme in the downlink in LTE-A/LTE-B based 5G systems, along with assistance for instantaneous links having harvesting capabilities for energy provisioning, need novel ideas of measurement for supporting required trials. For suitable RF measurements, trial equipment must automatically consider operating signaling protocols that utilize parameters defined by the user (such as a channel number). The UE operational testing should integrate the signaling protocol, handover testing, and end-to-end throughput. The main challenge faced in 5G UE testing is to guarantee that the response of state-change requirements is met [78].
- Dynamic heterogeneous resource optimization: It is difficult to endorse data transmission efficiency for the services getting URLLC as a top priority in mMTC. Due to the lack of radio resources, it is mandatory to consider their co-presence by combining their conflicting requirements and specifications concerning latency, reliability, density, and bandwidth. Therefore, the efficient arrangement of the resources in the wireless environment using dynamic and intelligent ways across various stages of service requirements is a demanding job [79].
- Efficient and realistic measurement: As data measurement is critical for the required modification/extension of current transmission models, the approach to measurement should cover various ranges of frequencies, spherical waves, 3D (elevation), and spatial consistency, along with new paradigms of communication, such as small cell and M2M/D2D communications. Furthermore, measurements must be captured for mm-wave (i.e., 60 GHz and over) for outdoor and indoor criteria, and they must feasibly apply to real-life scenarios (such as vehicle-to-vehicle/roadside communication, crowded areas, etc.) [80].
- Isolation among Network Slices: In a 5G network, many services have unique requirements. Consequently, the resources of a dedicated virtual network are required to certify the quality of service at every slice. A network needs high-performance slices isolated from each other. Through control plane and data plane isolation this isolation of network slices can be achieved. Generally, the slice control function can be distributed between various slices, whereas in some of the services, such as mission-critical communications, the resource sharing provides various benefits for infrastructure benefactors while it brings some challenging issues such as slice isolation. Network slices require control functionality. Moreover, the effective isolation of each network slice confirms that a security attack or any other failure does not alter another slice’s operation. Therefore, the mechanism of slice isolation is a predominant challenge while employing network slicing [81].
- Privacy protection: Obscurity services of 5G demands much more attention when compared with the previous cellular networks. The exclusive data rate of 5G carries a huge amount of data flow that contains private and sensitive information such as identity, private content, and position. In certain situations, the breach of privacy may lead to extreme consequences. For example, unintentional release of personal health data may expose the private information of a person, while the release of routing data for a vehicle may reveal its position to unauthorized others [82]. Due to the an application’s privacy requirements, the protection of privacy is a challenging issue faced by 5G wireless networks.
- Coordinated multiple points (CoMP): CoMP having 5G massive MIMO will play a critical part in enhancing the quality of communication, coverage, EE, and throughput of the network [83]. Moreover, mobile users can use relatively higher quality and better performance when located in another cell zone. Therefore, the CoMP system having 5G massive MIMO still has some open challenges—such as backhauling, processing, and cooperative framework—that demand more attention and study, in turn, to achieve maximum benefits of the network for the operator while keeping the cost in control.
- Deployment and Maintenance Cost: The expense of deploying and managing 5G is enormous. The industry has strict cost-cutting requirements, and new applications will only be deployed if they can be shown to save money over time [84,85].

### 5.2. Q2. What Is the Importance of Resource Allocation in 5G?

Resource allocation is an important aspect of wireless network systems. In a 5G communication network, it is important that the system be wiser and more dynamic to satisfy multiple network requirements. Power control, bandwidth allocation, deployment strategies, and association allocation are assigned resources in the system [86]. Resource allocation is an important aspect of any cellular network environment. It plays a significant part in maintaining friendly access for end-users, business partners, and customers of cellular-based applications. Resource allocation has great benefits for the cellular network environment. Network performance relies on the level of fairness of the network’s resource allocation. The fairness level has a strong correlation with the network’s performance level. The fairness levels of resource allocation are fair, perfect, unfair, and unbalanced. The levels of network performance are poor, less good, good, and perfect [87].

One main challenge in 5G is resource allocation as it relates to the performance of the long-life battery-powered devices and the service quality of the application. Users demand effective resource management and allocation. The ossified services and closed infrastructure of prevailing networks lead to inefficient and complex resource allocation. The existing network resources in wireless communication networks, especially the 5G wireless networks, are user-centric and demand effective resource allocation to acquire Quality of Services (QoS). Therefore, effective resource allocation is the main issue challenging the growing need for 5G cellular networks. A wireless communication network’s resources are specifically defined in terms of their power, spectrum, channel, etc., which must be allocated as per user requirements. A mobile network may suffer from spectrum resources shortage due to a massive increase in the number of users and in the number of devices connected to the network [88].

### 5.3. Q3. Which Current Policies, Strategies, and Algorithms Are Being Used for Resource Allocation in 5G?

This review paper classified the methods used for resource allocation in 5G based on a review of articles collected from various multiple sources. This systematic review explored the resource allocation algorithms and techniques executed by numerous investigators and organized them as per the methodologies that were employed in connection with any of the techniques. As per the inclusion and exclusion criteria, 71 research articles were selected for systematic study, as mentioned above in Figure 5. As shown in the Table 4 every article was studied from the perspective of the problem that was being addressed and the pros and cons of the techniques that were applied. In the current section, results are presented of our careful study of each strategy based on its ability to achieve effective resource allocation in 5G. The issues faced by 5G networks were resolved through hybrid models, swarm intelligence, genetic modeling, rule-based systems, and case-based reasoning. Resource allocation based on artificial intelligence techniques was utilized to resolve issues such as a hybrid problem-solving approach. The main goal behind the study was create a basis on which to select and/or improve a particular technique in the future as a means to allocate the resources of a 5G network.

In [89], the authors presented a supporting technique to handle the problem of interference, including co-tier interference and cross-tier interference, that distributed other tiers’ macro users that are associated with the users of that tier. The technique implemented an algorithm of online learning for effective allocation of a spectrum having modulation and adaptation of power competence. The simulation results illustrated the outperformance of the online scheme and attained a significant improvement in spectral efficiency, outage ratio, fairness, and throughput. The researchers examined the resource allocation for the downlink of two-tier heterogeneous networks containing macro-cell transmission having utilization of dual band and microwave frequency of small cells having millimeter wave and microwave frequencies [90].

Other authors discussed a novel approach for the base stations of a small cell with a dual band. The area covered by the small cell was categorized among two sections where the outer and inner section users were linked separately by small cells on microwave and millimeter wave frequencies. They designed a theory-based game approach having two layers for enhancing the spectral efficiency and energy efficiency of the system with the best utilization of existing radio resources. In [91], the authors presented GAPSO-PA (Genetic Algorithm Particle Swarm Optimization-Power Allocation), an allocation of power strategy that relies on the GAPSO algorithm. The GAPSO algorithm combines both GA and PSO algorithms like other swarm intelligence algorithms, and it is efficient for resolving the problems of non-linear optimization using cost efficient fast-global search. The resource configuration of the whole heterogeneous ultra-dense network was maintained through an SDN controller.

As per [92], the researchers discussed a unique algorithm of resource allocation (RA) for packet Universal Filtered Multi-Carrier (UFMC) BIC-based communications, which was applied in a unique modulation format in 5G wireless systems. The presented RA scheme enhanced the bit loading and coding rate among the overall bandwidth along with carrying per-sub-band power distribution. The researchers [93] produced useful D2D multicast links by sufficiently applying bot social and physical aspects of mobile manipulators, having the objective of enhancing the throughput of the whole social-aware network along with ensuring fair channel allocation among multiple D2D multicast groups. The proposed work primarily consisted of two segments, with the creation cluster having D2D multicast and jointly optimized allocation of both channel and power.

Evaluation results proved that, compared with a stochastic and heuristic algorithm, the proposed scheme enhance the entire social-aware network throughput by 50% and 5%, respectively. In [94], they presented a slice-based virtual resource scheduling having NOMA technology to increase the system’s quality of service (QoS). The authors formulated subcarrier allocation and power granularity allocation schemes into a Constrained Markov Decision Process (CMDP) problem, targeting improvement of the entire user rate. The above-used scheme further prevented the expectation calculation and the curse of dimensionality in the optimal value function. They developed and designed an adaptive resource allocation strategy that relied on Approximate Dynamic Programming (ADP) to solve the issue. The scheme could significantly improve the user data rate and minimize the outage probability.

As per [95], the authors presented an enhanced resource allocation low complexity algorithm for power allocation and user grouping optimization. The proposed model was designed to elevate system capacity. In this optimization model, the problem of complex non-convex optimization was split into two additional sub-problems that were separately solved in a step-by-step manner. Initially, all users were divided into groups using the greedy method, and later power allocation was executed on the sub-carriers of fixed groups. The results show that the presented scheme achieved better system capacity when comparing the existing algorithms and minimized complexity performance.

The authors of [96] initially introduced CRAN-based PSN architecture and modeled the problem of resource allocation on OFDM in C-RAN which relied on PSN allowing the tradeoff among projected allocation fairness bitrates of the PSN Service User (PSU). To overcome this, the resource allocation problem that had numerous variables was relaxed initially into one continuous variable that was solved using a proposed method that relied on Generalized Bender’s Decomposition (GBD). The authors utilized a Feasible Pump (FP) scheme to obtain a reliable numerical outcome for the real OFDM issue of resource allocation. The experimental results proved and highlighted that the maximum throughput attained with the proposed C-RAN relying on PSN was greater by 19.17% than the existing one relying on LTE. However, the average time for computation of the presented FP and GBD algorithms was less than Barrier by 51.5%, and GBD without relaxation was 30.1%, respectively.

According to [97], the authors presented a multitier architecture of H-CRAN that combined heterogeneous networks to work with a controller by forecasting the expectation of the user initially and then selecting the anticipated network depending on the user’s profile. A machine learning approach was considered to study the various network conditions and profiles having multiple pay load. The presented scheme was investigated under various conditions. The authors discovered that implementing a machine learning approach to C-RAN could provide an intelligent method for offering the selection of a network. The researchers in [98] focused on C-RAN slices for spectrum allocation of resources. They designed and developed a bankruptcy game-based algorithm to facilitate resources for the C-RAN slices. Slices and cloud were exhibited to the bankrupt company and defaulters in the game, respectively, where Shapely value was considered to acquire a suitable result.

The outcomes proved that the bankruptcy game-based algorithm prominently enhanced the utilization of resources while ensuring the allocation of fairness. The authors of [99] presented a unique nature-inspired wireless resource allocation approach having the distinctive observation of slices and which widely investigated the characteristics of slices and converted those slices into a model of profit for the utilization of network resources. Especially, the evolutionary interest relationships of users and personalized service preferences were utilized to portray the dynamic and complex network situation along with cellular automation, and a physically stimulated allocation approach of remote resource was presented as per the demands of user groups updated on regular basis. The outcomes mentioned that the presented approach attained desirable low computational complexity and resource utilization that supported the architecture of dynamic slicing of IoT while enhancing resource allocation flexibility and efficiency.

In [100], a VNF resource allocation approach based on context-aware grouping (VNF-RACAG) was presented, which empowered groups (depending on the environmental aspects of users, such as location and velocity) to evaluate the optimum groups to reduce the end-to-end delay of network services. Then, an algorithm based on graph partitioning was implemented to reduce the movement of the user among different groups, considering that the data rate lost by the users during VNF migration. As per [101], the researchers presented a hybrid decode-forward (DF)–compress forward (CF) approach that acquired the benefits of both DF and CF approaches in a receiver frequency-division relay channel (RFDRC). A nearby optimal resource allocation was proposed based on the DF–CF system, leading to a new reachable RFDRC rate. For the implementation, they additionally added a hybrid DF–amplify-forward (AF) approach and reconsidered the power allocation and data transmission rate.

Two positive outcomes were recognized when both relay and source frequency bands had an equal data transmission rate. The authors stated that the presented hybrid DF–AF approach could attain the concave envelope of the maximum concerned DF and AF rate. The presented approach brought significant improvement to the RFDRC. According to [102], they delivered device-to-device (D2D) modes 3 and 4 for the communication of automobiles over Sidelink (SL). The authors presented a summary of the scheduling and resource allocation mechanisms with an emphasis on the comparison among modes 3 and 4. They addressed the foremost differences with modes 1 and 2, with a focus on addressing the reliability and latency requirements by utilizing improved scheduling and resource allocation, respectively.

A simulation was performed to attain the results for evaluating the SL D2D performance of modes 3 and 4 with respect to collision probability and Block Error Rate (BLER). In [103], they presented a framework for simultaneous wireless information and power transfer (SWIPT). Initially, the authors described the actual energy efficiency and actual capacity of SWIPT in multi-user orthogonal frequency division multiple access systems by optimizing the hands-on approach of SWIPT: power splitting (PS) and time switching (TS). Later, they investigated two problems of resource allocation to enhance the actual energy efficiency and capacity separately by focusing on three attributes: QoS delay, average sum transmission power, and minimum harvested power. The obtained calculation demonstrated that there was a basic tradeoff between the performance and harvested power relative to effective energy efficiency and capacity.

As per [104], the authors proposed a resource allocation system for D2D users. Using the proposed system, the related pairs of D2D users could utilize their resources themselves by following three steps. First, the objects sustained their occupancy matrix of resources by interchanging data from neighboring devices. Second, based on the resource allocation criteria, a resource block was created. Finally, the resource block was allocated depending on BS side priority. The presented system relied to a lesser extent on the side base station. Therefore, it minimized the BS side workload, along with minimizing the consumption of time in the process of resource allocation. In [105], the researchers presented an algorithm that depended on the distribution concepts by which interference management and resource allocation are completed at each tier level while depending on the locally available information.

The results available after the simulation evaluated performance based on comparison of the existing work with the presented algorithm with respect to the average data rate and efficiency of resource allocation attained for each user. According to [106], the authors presented a novel cross-layer infrastructure for resource allocation and downlink scheduling that was able to exemplify the characteristics of two of the most favorable waveform technologies with respect to 5G networks—namely, orthogonal frequency division multiple (OFDM) and filterbank multicarrier/offset quadrature amplitude modulation (FBMC/OQAM). Unlike the comparative analysis discussed in previous literature, the publication of these two abovementioned technologies were highly centered on PHY metrics.

The presented work reveals the extraction of user-relevant metrics such as average delay, throughput, service coverage, and Jain’s fairness index in a multi-class traffic environment. In [107], the authors emphasized relay selection and downlink resource allocation, where a user is coupled through a multi-hop relay to a base station while considering various relay stations for the purpose of selecting one that is in his range. To tackle the supplementary issues presented by multi-hop relay nodes, they proposed a scheme for dynamic resource allocation and the choice of relay. A mathematical analysis was portrayed to illustrate the validity of the presented scheme. The authors of [108] presented a small-complexity technique based on subgroups that attained maximum performance. The outcome was appropriate for employment in hands-on systems, such as Satellite Long-Term Evolution (S-LTE), as the computational cost did not depend on the number of available resources and the multicast group size.

The effectiveness of the presented technique was examined through simulations performed in various multicast group and radio transmission environments. In [109], the authors presented an end-to-end slicing that acted as in the capacity of providing a computing and communication resources system over the entire 2-tier architecture of edge computing having multi-access. This system was deployed by utilizing open-source tools. It was observed that the presented framework significantly incorporated the resources of 5G, which were either communication or computing among slices and guaranteed that the implemented slices’ resources were more efficient in achieving the tenant’s latency requirements. Furthermore, the experiments revealed that mMTC and URLLC services needed a supplementary 70% of the required communication and computing resources delivered by the RAN and edge to comply with their stricter latency requirements.

As per [110], the authors introduced a unique resource allocation scheme that was named a threshold-controlled access (TCA) protocol, in which an uplink resource allocation scheme through which the device itself made decisions to allocate blocks of resources depending on the relevant application’s power profile and battery status ultimately led to attaining a favorable QoS metric. At first, the presented TCA scheme chose multiple carriers for the purpose of allocating resources to a specific node for improving the life of MTC devices having reduced consuming power. Subsequently, the well-organized solution was deployed by pursuing a threshold value. The specific value was considered to select via plotting the QoS metric. The threshold improved the subcarrier selection for reduced-power devices such as small e-health sensors.

In [111], the authors presented a communication framework to subtenant the implementation of a CPIoTS having a central controller. Using this framework, various actuators and sensors could create communication links in full duplex mode along with the main controller. To deal with the available band signal data, the problem of resource allocation was considered as non-convex mixed integer programming problem, focusing to enhance the CPIoTS total energy efficiency. By proposing the alteration, they divided the issue of resource allocation among channel and power allocations. Furthermore, the authors considered an energy-efficient allocation of power scheme depending on game theory and Dinkelbach’s algorithm. Consequently, to minimize the computational complexity, the model of channel allocation was designed similar to a 3-dimensional matching problem and was resolved through an iterative Hungarian method with virtual devices (IHM-VD).

In [112], the authors proposed a consolidated resource allocation system using online learning, which maximized energy efficiency and ensured interference mitigation while sustaining the requirements of QoS for every user. To find the better effectiveness of this system utilizing model-free learning, they considered the priority of users in a compact state representation-based resource blocks (RBs) allocation learning method to improve the learning process. The results revealed through simulation showed that the presented solution of resource allocation could alleviate interference and enhance both spectral and energy efficiencies significantly while maintaining users’ QoS requirements. The authors of [113] presented an underlay device-to-device resource allocation utilizing an outdoor mmWave situation. The authors focused on the fair allocation of resources in a cell to enhance the spectral efficiency.

They also discussed cellular and ad hoc communication while utilizing the same strategy depending on the requirements. The problem was mathematically illustrated to enhance the total rate and resource allocation approach that was proposed. The purpose behind this work was to achieve maximum system capacity using the presented resource allocation strategy, which did not have exclusivity for resource allocation and allowed multiple users to utilize the same resource block without degrading the system’s spectral efficiency. The authors [114] proposed an algorithm that they named tri-sage fairness (TSF) to overcome the issue of resource allocation in an ultra-dense network (UDN) that had caching and self-backhaul, through which cells without a direct network connection (rTP) could contact the core network through a donor TP (dTP).

In TSF, the rTP considered whether to transfer files cached in rTP (rTP files) or the files not cached in rTP (dTP files) based on link capacity and delayed and allocated access link resources using a proportional fairness algorithm. The dTP allocated backhaul resources among rTPs and its users with fairness considerations and decided the time each rTP spent on the backhaul link. Complexity, overhead, efficiency, and fairness were mutually achieved in TSF. In [115], the authors considered the capacity of fronthaul, in which the controller enhanced the time and average network throughput by implementing a Coarse Correlated Equilibrium (CCE) and incentivizing base station (BSs) to optimize decisions for ensuring mobile user’s (MUs) quality of service requirements. By utilizing tools from game theory and Lyapunov stochastic optimization, they presented two time-scale methods in which the controller provided recommendations—e.g., subcarriers having least interference—while in long time-scale, BSs manage their MUs, and available resources are allocated in each time slot.

The authors of [116] discussed the Nakagami-m model, and they launched an MIMO-OFDMA relay-based cognitive radio network. By providing the diverse numerical attributes of QoS, they analyzed and derived accumulated effective capacity using their established resource allocation policies along with MIMO-OFDMA-based cognitive radio networks. The researchers [117] aimed to enhance simultaneously the energy and spectrum efficiencies of UDN and ensure the macro cell QoS by proposing combined allocation of RBs and transmission power. To balance the adjustment of these two conditions, a multi-objective optimization problem (MOOP) was designed that optimized SE and EE jointly. In addition to an outdated weighted sum method which could not provide optimal SE and EE simultaneously, an enhanced NSGA-II based resource allocation algorithm was presented.

In [118], the authors investigated using three promising technologies that were power domain non-orthogonal multiplane access (PD-NOMA) and had coordinated multi-point transmission (CoMP) and dual connectivity. The primary purpose was to enhance the downlink energy efficiency (EE) by utilizing both microwave and millimeter wave links in access and fronthaul, while engaging CoMP and PD-NOMA. In this manner, a heterogeneous cloud radio access network (HCRAN) for downlink was utilized by joint fronthaul and access radio resource allocation. The authors of [119] investigated the computing resource allocation and joint communication along with baseband unit (BBU), user association, and remote radio head (RRH) in C-RANs. First, they established a model based on queue in C-RAN; second, they created a formula of both optimization problems for computing (such as virtual machines (VMs)) and communication (such as power and resource blocks (RBs)) resource allocation, aiming to minimize the mean response time.

Queueing stability constraints, interference, and user association with RB allocation were observed in the communication resource optimization challenge. The computing resource optimization issue considered VM allocation and BBURRH mapping for SCs, controlled by queuing stability and BBU server capacity. To overcome the computing and communication resource optimization challenge, they presented a combined resource allocation solution that utilized the double-sided auction-based distributed resource allocation (DSADRA) method, in which users and small cell base stations mutually contributed using the auction theory concept. In [120], the authors presented a resource block (RB) and combined selection of allocation for device-to-device D2D communication using a wireless network. They discussed the interference across different D2D links working the edge of surrounding cells. D2D communications offer a consistent transmission of the neighboring cell edge while interference—partially from CUs and D2D pairs—belongs to the edge of neighboring cells.

According to [121], various issues occur when end-to-end (E2E) slices are rapidly deployed on the infrastructure of the network due to complicated infrastructure qualities of the backhaul transport network. First, they presented a paired decision resource allocation model in which they initially articulated a paradigm for mapping relationships in a synchronized way among substrate networks and logical networks. They defined the latency optimal virtual resource allocation issue to enhance the user experience and improve quality of service, which was corelated with bandwidth constraints and backhaul capacity. The issue was specified as integer linear programming (ILP) and overcame the use of the branch and bound scheme, which produced a traffic routing policy and optimal virtual network function (VNF). In [122], the authors proposed the allocation of resources for their estimates of highly remote positions by using machine learning.

Specifically, they used the supervised ‘random forest’ machine learning technique for the designing a learning-based method of resource allocation by exploiting the behavior of a user’s location estimates and system parameters. Through this, the CSI acquisition overhead was sidestepped by utilizing the location estimates that had better utilization of the spectrum. The authors of [123] presented a resource allocation scheme having the least interference in 5G cellular network with hop D2D communications to purposefully reduce interference. First, this scheme calculated BS interference across every resource block on the destination and relay side. After calculation, the resource block was allocated to those having less interference among all blocks. The BS gave high priority to those blocks that created the least interference. The obtained results provided better performance compared with other random resource allocation algorithms.

The researchers in [124] proposed IoT resource allocation and multiband cooperative spectrum sensing in cognitive 5G networks. The multiband scheme minimized energy consumption for the spectrum compared with other single-band approaches. They developed an optimized approach for obtaining the least number of sensing channels at every node of IoT in the multiband scheme to reduce the consumption of energy for detecting the spectrum while substantially increasing the detection possibilities and requirements for an incorrect alarm. The presented CRLS effectively satisfied QoS requirements for resource allocation by spectrum access. The authors of [125] presented a solution with reduced execution-time similar to CTA-PSO, proving the implementation appropriateness in a wireless mixed multimedia environment. To justify the increasing requirements of new applications in a high capacity and converged network such as 5G, other techniques of resource allocation such as CTA-PSO must be additionally examined.

Ref. [126] presented a multi-objective scheme of resource allocation for a density-aware virtualized software-defined cloud radio access network (C-RAN) considering a two-design RAN-based mode for typical density users: low-density and dense region mode. The limitations of fronthaul capability were undertaken separately through the data plane and control plane, which was more crucial in the dense region. The results showed that complete centralized process and management and efficient energy utilization of structure in a short amount of traffic time were accomplished by turning off data RRHS. The authors of [127] presented the probabilistic characterization of the feasibility of 5G slice resource allocation issues to determine whether or not they could be addressed. They presented a mini slot-based slicing allocation (MISA), which is a unique spectral-efficient scheme to assign PRBs for the URLLC and eMBB service-based utilization of mini-slots.

They studied the Wang–Landau algorithm to illustrate the acceptability of the limitations to avail the transition segment that segregates feasible and infeasible slice rate areas. The presented scheme enhanced the spectral efficiency regarding the single slot-based model. The researchers [128] discovered the deterministic mechanism of resource allocation to fulfill URLLC characteristics regarding latency and reliability, consisting of initial transmissions and controlled retransmissions. A joint coding and modulation scheme for resource allocation was executed to reduce resource consumption, to benefit reliability and latency. They presented the results of the proposed technique while achieving the lowest error rate. The authors of [129] presented a cross-layer D2D link control framework ensuring QoS and enhancing video streaming QoE having various delay and priorities limitations. The authors discussed three techniques in this framework, consisting of flexible communication mode switching UE, priority-based video transmission, and subset-based assignment of relay.

The proposed technique significantly achieved good results regarding average energy consumption, average peak signal-to-noise ratio (PSNR), and average mean time to failure (MTTF). In [130], the authors used the random forest algorithm for developing a learning-based allocation of resources that facilitated multiple user terminals utilizing their location data. The presented scheme worked with more complexity and lower system overhead as compared with a CSI-based resource allocation approach. It also additionally demonstrated significant or comparable system performance with a CSI-based approach for multiple user densities in the system. As per [131], the authors investigated the research and background challenges of D2MD content sharing in social-aware cellular networks and proposed a D2MD content sharing approach. In this approach, social and physical domain factors were examined to provide geometry programming and efficacious clusters; bipartite matching was used to obtain channel assignment and power control for the delivery of shared content. The results showed considerable enhancement of throughput.

The authors of [132] examined the problem of resource allocation in H-CRAN with a downlink having D2D communication, in which various RRH users (RUEs) and RRH were permitted to reuse a subchannel that was already assigned to MUE. The resource allocation problem was articulated as a mixed integer nonlinear programming (MINLP) problem that was NP-Hard. To overcome this issue, rearticulation was performed as an external many-to-one game followed by a coalition game. Then a coalition formulation and constrained DA algorithms were presented to provide the solutions to these games, separately. The complexity and stability of these algorithms were tentatively achieved. Depending on the discussed two methods, the outcome of the presented algorithm was achieved successfully. The simulation outcomes confirmed the usefulness of the presented algorithm related to fairness, admitted users, and throughput.

According to [86], the authors presented a unique resource allocation approach (hybrid resource management) to address the issue of EE maximization in scenarios of wireless networks—i.e., cell-free, massive MIMO HetNets, massive MIMO and small cell. In addition to this, the important constraints of power budget and QoS threshold were ensured while the objective EE function related to bits/Joule/Hz was improved. In [133], the authors examined resource allocation in a scenario involving the automation industry, where the main dominant controller focused on transmission of various packets to two selected objects (such as an actuator and a robot). In this scenario, two transmission approaches are examined: relay-assisted transmission and orthogonal multiple access (OMA). The authors jointly examined the power allocation and block length to reduce the actuator’s error probability related to the robot’s reliability requirement and latency constraints. As per [134], the authors aimed to find the power control and optimum user association schemes for energy efficiency enhancement with system’s QoS constraints.

The authors proposed a distributed algorithm. They first discussed the solution for providing optimum user association for static transmission power. Additionally, user association optimization and a joint power control scheme was studied by investigating load in energy-cooperation enabled NOMA HetNets, which attained higher performance in accordance with energy efficiency compared with existing approaches. Authors of [135] focused on overcoming the presented optimization issue; the authors utilized a worst-case strategy by redrafting the issue from the perspective of the protection function to gain a better understanding of supplementary manageable design. Afterward, they implemented the alternate search method (ASM) in which every repetition beamforming, user association, and cooperative codebook allocation subproblem was resolved individually by continuing the algorithm until achieving convergence. The mathematical findings proved that the presented optimization issue through MISO and SCMA technologies boosted the system efficiency significantly, even for indeterminate CSI.

The researchers of [136] proposed an effective resource allocation method utilizing online learning, which enhanced energy efficiency and mitigated interference while managing the requirements of QoS for every user. The resource allocation consisted of power and resource blocks (RB). The proposed method was integrated through centralized and decentralized tactics. In the centralized approach, RA was handled at a unified organizer having the baseband managing unit, while in the decentralized tactic, macro-BSs cooperated to attain the best resource allocation. The results illustrated that the proposed scheme of H-CRAN enhanced and maintained energy efficiency and users’ QoS, respectively. The researchers [137] presented a unified iterative resource allocation scheme that could distribute power and RB jointly. The system expanded the femtocell throughput while sustaining the restrictions of cross-tier fairness and interference of assorted amenities. By altering the feasible domain and variables, the main challenge was converted into the standard convex optimization form which could be addressed by the Lagrange duality method.

The authors [138] proposed hybrid MC-NOMA systems based on a joint resource allocation scheme that was relevant to a generalized scenario having an identical subcarrier for various users that could be multiplexed. For each user, they investigated the fewest number of requirements that existed simultaneously in the system and that could provide significant influence on the OMA and NOMA selection. The hybrid MC-NOMA mode considerably beat both OMA and NOMA related to the EE–SE tradeoff, and it also displayed excessive potential to progress the tradeoff between user system efficiency and fairness. The researchers in [139] investigated energy efficient resource allocation in a 5G challenge having soft frequency reuse (SFR). The power allocation and RB assignment were optimized jointly under the umbrella of SFR. The Stackelberg game model was presented to acquire the maximum EE in the 5G network under inter-cell interference (ICI), and to limit the ICI, the interference pricing factors were utilized along with the authors provision of the NE point’s existence.

Due to non-convex object function, they used the Lagrange duality method of decomposition to achieve the ideal outcome for the power allocation challenge. By using several iterations, they achieved the maximum energy-efficient resource allocation for the 5G network. The authors of [140] aimed to support the maximum number of those who could access the system simultaneously; the authors proposed a virtual code resource allocation (VCRA) method that extended the code-expanded approach. Furthermore, they introduced the virtual resource allocation method to ensure energy-priority in the access technique. The main purpose was to elaborate the various access levels that meaningly split a cluster of access codewords, appropriately maintained to ensure maximum capability for every access level. The authors of [141] proposed matching slice architecture for resource allocation based on the idea of a self-organizing network.

The running architecture initially shaped the processes and functions of the matching independent management of the resource. Based on the multidimensional statistics, an efficient deep learning model named LSTM (long short-term memory) was utilized to build the dynamic multicast service traffic model in space–time, which facilitated the base for more network resource allocation. By relying on the obtained results and satisfactory conditions of users changing requirements, the corresponding model was developed to minimize the RRHS energy usage and to maintain QoS as constraints. In [142], the authors proposed an innovative deep strengthening learning-based intellectual Time Division Duplex (TDD) configuration system to dynamically allocate radio online resources. They deployed a deep neural network to obtain the characteristics of complex network information, and the dynamic Q-value iteration-based strengthening learning along with an experience replay memory mechanism was presented to adaptively change the TDD Up/Down-link ratio based on estimated rewards.

They obtained significant network performance enhancement with respect to both packet loss rate and network throughput. As per [143], the authors employed DRL to develop an optimal resource allocation and computation offloading scheme for reducing the energy consumption of the system. Initially, they discussed a multi-user end-edge-cloud composed system in which all base stations and devices had computation proficiencies. In the next step, they investigated the joint resource allocation and computation offloading problem as a Markov Decision Process (MDP) and presented a new DRL scheme to reduce the system’s consumption of energy. The results obtained by using a practical dataset illustrated that the presented scheme provided excellent performance to achieve the required goal.

In [144], resource allocation for multi-users in a 5G massive-MIMO (mMIMO) was executed through a deep neural network (DNN). In the first phase, the unbiased functions were enhanced through the Multi-objective Sine Cosine algorithm (MOSCA). The unbiased functions that were observed through the optimization method were energy efficiency (EE), power consumption, signal-to-interference-and-noise ratio (SINR), and dataset. In next phase, these unbiased functions were assigned to a neural network for the allocation of resources. The DNN recognized the level of requirement for all users. Depending on this level status, resources were allocated to every user by maintaining EE and high throughput. Moreover, the fairness level of the neural network-based resource allocation process was also recognized.

The researchers of [145] proposed a scheme for categorizing resource allocation into two main parts named as resource allocation and medium access. The medium access influences the transmission nature of the wireless signal and MTC devices’ wait time to allocate priorities utilizing capillary band in an integral way. Meanwhile, in resource allocation, SNR, whole induced transmission-awaiting, and transmission delay MTC devices were considered to allocate resources in the cellular band. The reflection of two-staged dynamic priorities in the proposed scheme brought significant performance enhancement in outage and success probabilities. The researchers in [146] proposed a scheme to minimize interference for 5G cellular users (Sus) that was focused on interference threshold, minimal transmission rate, available power, and quality of service (QoS). At first, the mandatory least transmission power by the V2X users (VUs) was assigned as the initial power value.

Next, the Hungarian algorithm was performed to acquire a suitable subchannel. Finally, an approach for optimization was presented to the power allocation. The findings revealed by simulation illustrated that the presented method confirmed the smallest transmission rate of VUs, and it enhanced the CUs’ channel capacity while ensuring the QoS of the CUs. The authors in [147] addressed the dynamic latency-aware resource allocation problem in multi-tenant 5G slice networks, a multi-tier heterogeneous environment, for efficient radio resource management. The problem was expressed as a higher utility optimization problem. The optimization problem was altered, and a classified decomposition method was implemented to decrease the complexities while solving the optimization problem. Additionally, the authors proposed a genetic algorithm (GA) intelligent latency-aware resource allocation scheme (GI-LARE). Authors compared GI-LARE with static slicing (SS) resource allocation, the bound-based scheme and spatial branch, and an optimal resource allocation algorithm. The results revealed that GI-LARE outperformed the other mentioned schemes.

As per [148], the authors proposed a centralized low-complexity packet scheduling scheme to provide URLLC QoS services. Progressive 5G NR system-level results were discussed to evaluate the effectiveness of the presented scheme. It was observed that unified architecture improved URLLC latency. Compared with effective point selection and scattered scheduling for dynamic spectral, the presented scheme attained a 99% and 90% reduction in latency for URLLC, respectively. The authors of [149] developed an arrangement technique inside the IoT traffic to offer end-to-end QoS in an NB-IoT network. They established a process for handling a smart queue based on the IoT traffic arranging processes. Through the many simulations, they verified that the developed method guaranteed high E2E QoS of the present traffic. This was accomplished through decreasing an average E2E communication delay of the real-time messages.

The authors in [150] obtained their outcome in two phases. In the first phase, numerous VUEs were released from unlicensed frequency bands and the time factor of the duty cycle scheme, while in the second phase, the issue was transformed into a convex optimization problem, which was resolved through the presented Lagrange duality method (LDM). The simulation findings expressed the performance of the presented application scenario having a Wi-Fi or LTE system. Furthermore, the presented scheme performed efficiently related to throughput, along with ensuring QoS of WUEs as compared with the general greedy algorithm. In [151], the authors focused on the resource constraints, and based on 5G enabler concepts and operative bandwidth, a resource allocation scheme was presented that could achieve the requirements of reliability and delay for URLLC traffic.

Latency components and end-to-end error were presented and the interchange existing between the error components was used for minimizing data rate. A unified queuing strategy, time frequency, and packet delivery resource allocation for CoMP empowered URLLC in C-RAN architecture were presented. The presented system illustrated the efficient performance regarding UE satisfaction and resource utilization compared with current techniques. In [152], the joint optimization issue was examined along with ambiguous channel rising to achieve maximum energy efficiency and reducing intra-cell interference. The likelihood limitation was converted to the deterministic one based on the fundamental conversion. By using successive convex approximation and a relaxation variable scheme, the novel integer non-convex optimization challenge was shared by two resolvable convex sub-challenges. The power control and user association algorithms were focused to fix the optimal resource allocations. The findings revealed by simulation illustrated the effectiveness of the scheme and were shown to be robust in the dynamic communication environment.

In [153], the M/G/1 queuing model was deployed to investigate the inaccurate transmission retrieval interruption of URLLC multiuser amenities, and in applying this model, the lowest essential data rate was designed and implemented on an adaptive control scheme. The presented Pollaczek–Khinchine (P-K) formula-based quadratic optimization (PFQO) method for controlling the maximum retransmission parameter of the hybrid automatic repeated request (HARQ) technique in URLLC enhanced the bandwidth requirement. The findings revealed in a simulation displayed the bandwidth saving effect of the presented PFQO scheme based on various packet length distributions and signal-to-interference-and-noise ratios (SINRs).

In [154], the articulated optimization problem was related to the mixed integer nonlinear programming (MINLP) problem, which is NP-hard and which needs a comprehensive search to obtain an ideal result. Nonetheless, the computational complexity of the comprehensive search increased exponentially with the growth in the number of users. Hence, an outer approximation algorithm (OAA), having least complexity, was presented to attain a close-to-optimal solution. Wide-ranging simulation exercises were conducted to assess the proposed system. Outcomes focused on the usefulness of the projected innovative decoupled cell association scheme over the traditional coupled cell association scheme regarding mitigating interference, users attached/associated, offloading traffic to address sum–rate maximization, and traffic imbalances.

The researchers in [155] proposed a resource allocation scheme that addressed network slicing by applying the Powell–Hestenes–Rockafellar technique and the branch and bound system, obtaining an ideal result. The outcomes proved that the proposed resource allocation scheme could significantly enhance URLLC spectral efficiency and the system’s reliability, in contrast with the equal subcarrier allocation (ESA), the equal power allocation (EPA), and the adaptive particle swarm optimization (APSO) algorithms. Moreover, the authors investigated the algorithm’s spectral efficiency associated with the modification of users’ requirements for two slices, and it achieved better spectral efficiency performance. The researchers [156] presented a utility function based on the signal-to-interference-and-noise ratio (SINR).

The small cells quantity in a cluster demonstrated the weighted mean. From all clusters, a small cell was chosen as having an extreme value for the second utility function based on the lowest path loss values across the small cells and the microcell base station. The small cell having a high priority performed similarly to a spectrum manager of a set. The remaining small cells presented a price value created based on the required data rate and the user type for a subcarrier to the high priority small cell spectrum manager. The small cell that had a high priority assigned resources to SCs which were being used for the projected algorithm relying on the third utility function along with the price value. In the presented work, they calculated the spectral efficiency, SINR, and power consumption of the system. The power consumption of the presented system was reduced by up to 30%, and spectral efficiency and SINR improved almost 40% and 45% compared with corresponding existing methods.

The authors of [157] proposed an effective resource allocation method for 5G C-RAN named Bee-Ant-CRAN. The problem discussed was to develop joint mapping logically among User Equipment (UE) and RRHS along with BBUs. This was tested under network load circumstances, aiming to minimize the overall costs of the network along with managing the QoE and QoS of the user. The load was articulated as a mixed integer nonlinear problem with several restrictions. Afterward, the expressed optimization problem was classified as a two-step problem of resource allocation: RRH-BBU mapping and UE-RRH association.

In [158], the authors proposed a game theory-based ideal method for resource allocation, which focused on enhancing the coverage probability and sum rate for uplink communications in critical scenarios. The presented classified game theory architecture improved the performance of a multitier heterogeneous network having uplink communications in alliance with femto access points and pico base stations in the domain of a macro base station. The experimental simulations were based on a real-time data set that was being observed for a predefined period. Then the data set was deployed to generate real-world critical scenarios. The result was achieved by using a Nash equilibrium strategy for a noncooperative game. The authors performed simulations that had various failure rates, and the outcomes showed that the presented method enhanced the sum rate coverage probability, obtaining a remarkable margin with or without considering the critical scenario.

### 5.4. Q4. Which Metrics and Parameters Are Considered during Resource Allocation in 5G?

As shown in the Table 5 the following are the metrics that were found to be used in 5G resource allocation in this review.

**Figure 8:**
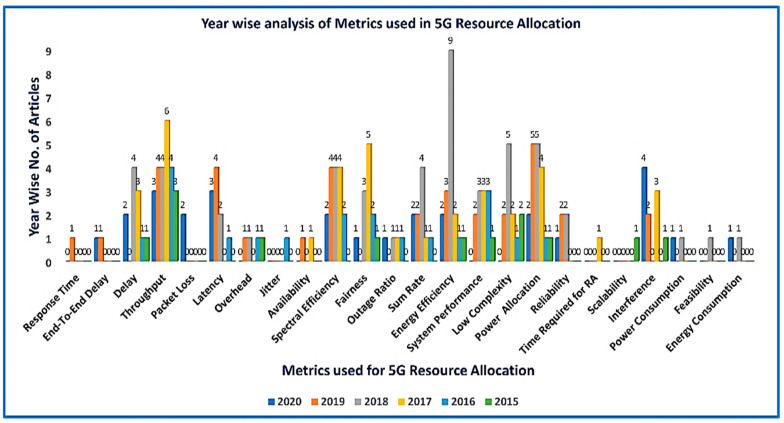
Year-wise analysis of metrics used in 5G resource allocation.

The metrics used in the literature reviewed in these articles are considered as packet loss, throughput, delay, latency overhead, jitter, response time, availability, spectral efficiency, fairness, outage range, sum rate, energy efficiency, system performance, low complexity, end-to-end delay, power allocation, reliability, the time required for resource allocation, scalability, interference, power consumption, feasibility, and energy consumption. The year-wise analysis of metrics that were used in this extensive literature are shown in Figure 8. This figure illustrates the total number of articles in each year. In 2015, the metrics used for resource allocation in articles were: delay = 1, throughput = 3, overhead = 1, fairness 1, energy efficiency = 1, system performance = 1, low complexity = 2, power allocation = 1, scalability = 1, and interference = 1. In 2016, the metrics used in the articles were: delay = 1, throughput = 4, latency = 1, overhead = 1, jitter = 1, spectral efficiency = 2, fairness = 2, outage ratio = 1, sum rate = 1, energy efficiency = 1, system performance = 3, low complexity = 1, and power allocation = 1. For the year 2017, the metrics used in the articles were: delay = 3, throughput = 6, availability = 1, spectral efficiency = 4, fairness = 5, outage ratio = 1, sum rate = 1, energy efficiency = 2, system performance = 3, low complexity = 2, power allocation = 4, and time required for RA = 1. For the year 2018, the metrics used in the articles were: delay = 4, throughput = 4, latency = 2, overhead =1, spectral efficiency = 4, fairness = 3, outage ration 1, sum rate = 4, energy efficiency = 9, system performance = 3, low complexity = 5, power allocation = 5, reliability = 2, power consumption = 1, feasibility = 1, and energy consumption = 1.

In the year 2019, the metrics used in the articles were: response time = 1, end-to-end delay = 1, throughput = 4, latency = 4, overhead = 1, availability = 1, spectral efficiency = 4, sum rate = 2, energy efficiency = 3, system performance = 2, low complexity = 2, power allocation = 5, reliability = 2, and interference = 2. For the year 2020, the metrics used in the articles were: end-to-end delay = 1, delay = 2, throughput = 3, packet loss = 2, latency = 3, spectral efficiency = 2, fairness = 1, outage ratio = 1, sum rate = 2, energy efficiency = 2, power allocation = 2, reliability = 1, interference = 4, power consumption = 1, and energy consumption = 1.

**Figure 9:**
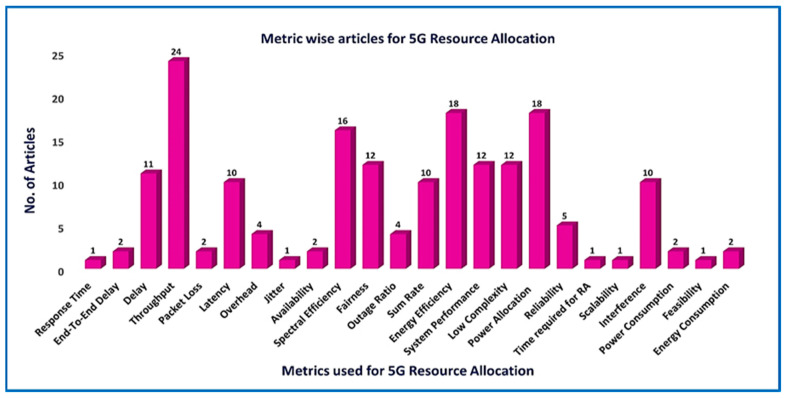
Analysis of metrics used for 5G resource allocation.

In this extensive systematic review, we noticed that the metrics used by researchers in multiple research papers were totaled: response time, 1; end-to-end delay, 2; throughput, 24; packet loss, 2; delay, 11; latency, 10; overhead, 4; jitter, 1; availability, 2; spectral efficiency, 16; fairness, 12; outage range, 4; sum rate, 10; energy efficiency, 18; system performance, 12; low complexity, 12; power allocation, 18; reliability, 5; the time required for resource allocation, 1; scalability, 1; interference, 10; power consumption, 2; feasibility, 1; and energy consumption, 01; as shown in Figure 9.

Table 6 illustrates the domains used for this literature review consisting of fronthaul, C-RAN, H-CRAN, backhaul.

**Figure 10:**
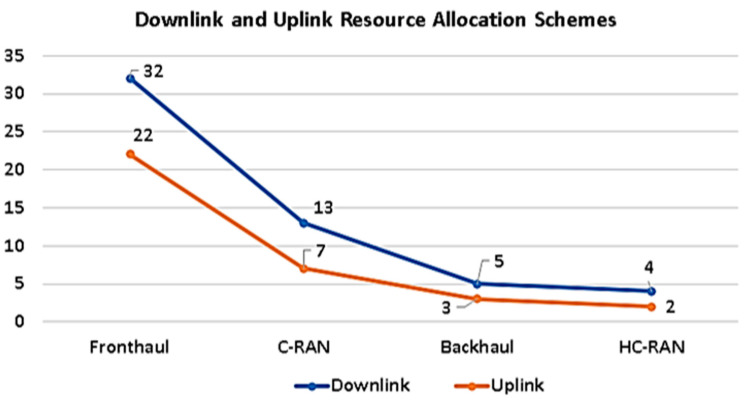
Articles studied for downlink and uplink resource allocation schemes.

Figure 10 presents the number of papers reviewed in this extensive literature review that discussed downlink and uplink resource allocation from the perspective of fronthaul, C-RAN, backhaul, and HC-RAN. The number of reviewed papers was 32 for fronthaul, 13 for CRAN, 5 for Backhaul, and 4 for HC-RAN for downlink schemes while the uplink schemes papers reviewed were 22, 7, 3, and 2, respectively.

### 5.5. Q5. Which Open Issues and Research Trends Were Unaddressed in Resource Allocation in 5G?

Because of the evolutionary enhancement in IoT and data requirements, the entire wireless communication system has been completely altered, such as in M2M communication or V2V networks. RA is still facing enormous challenges at each level. Therefore, many challenges such as communication security, network infrastructure, spectral efficiency, and energy efficiency need to be addressed by researchers in the near future. For efficient next-generation communication in the future, it is observed that some main challenges such as lifetime operation networks and the benefits of green communication for the goal of saving energy will be a challenging task. For example, the issues related to resource allocation for achieving energy harvesting networks and green communication networks will receive substantial attention in the near future.

It is observed that spectral resource (SR) is a limited resource and precious for wireless communication. Therefore, there is a need to develop some useful methods for enhancing the SE. Dynamic RA and spectrum detection capacity are significant issues in resource-sharing cognitive networks. Additional new issues for RA in various cellular networks (CNs) may also arise.

Networks will certainly move towards the development of more powerful functions, higher data rate, better transmission efficiency, and so on from the perspective of network structure. Due to this, how to achieve multiuser diversity optimization and joint antenna selection for multiuser MIMO networks is a challenging issue. Since both ultra-intensive users and multi-antenna systems are a developing trend, the scarce SR and limited bandwidth bring several challenges for the structure and practical application of RA in cellular networks.

Information security is essential for a communication system from the perspective of information transmission, specifically in cellular networks. Even though CNs can acquire multinetwork integration and fulfill various user requirements, there may be security problems, eavesdropping situations, and information leakage. As a result, RA for consideration of security limitations is essential in multiuser CNs owing to the complex communication scenarios, such as RA for physical layer security in CNs.

RA has various problems for various application situations. The optimization problem can be a multivariable one. Our focus was to obtain computation offloading and caching optimization in the communication system. In this way, we focused on the complicated and practical application environment. Moreover, from the solution process perspective, self-optimization and more intelligent algorithms should be introduced and developed for upcoming future CNs, such as machine learning for wireless communication applications. The related challenges can be matched adaptively by these machine learning algorithms. The training system can dynamically adjust its parameters of optimization to address the wireless network’s requirements. The RA challenges in CNs will attain better solutions in the future by using these intelligent algorithms.

## 6. Open Research Issues and Trends in 5G

There are still some areas that need to be explored by researchers. Here, some of the open issues are discussed below:

### 6.1. Joint Resource Allocation Techniques

Sophisticated and advanced allocation schemes are needed broadly due to the requirement of additional computing resources. One main challenge is to develop resourceful compression algorithms for fronthaul links. From this end, it is essential to measure and analyze the latency effect on the upper layer’s performance of the fronthaul. Moreover, optimal resource allocation in contexts of constrained fronthaul requires more investigation. Fronthaul links that experience packet loss can be one more thought-provoking topic. The fronthaul network is predictably extremely diverse and has latency and various link capacities, which necessarily demand re-configuration of fronthaul so it can be altered based on traffic load and network topology.

### 6.2. Fronthaul/Backhaul/C-RAN Issues

The performance achieved in sum-rate can be enhanced by using the adaptive before/after-precoding method. For this purpose, it is essential to measure and analyze precoding problems that use minimum backhaul. Similarly, the users’ accurate profiling is a important breakthrough when examining suitable approaches for the development of backhaul re-configuration in CRAN. Furthermore, effective algorithms need to be developed to increase the performance of the existing system depending on traffic load and user profiles to evaluate the optimal backhaul.

Additionally, BS performance investigation with clustering (specifically having large size clusters), while keeping in mind the reconfigurable backhaul ultra-dense BSs deployment, will likely be an auspicious research gap in the future. Furthermore, the study in this domain should emphasize examining effective resource optimization methods by keeping in mind the limitations of both backhaul and fronthaul links while considering the user-side demands.

### 6.3. Minimization of Latency

The number of transmission delays may be elevated by increasing the number of BSs. It is essential to inquire about the scheduling delay and effect of transmission, as these can particularly contribute to the proposed schemes for real-time processing capability. It is also essential to discuss the trade-off between delay and performance triggered by coding across multiple-fading blocks.

### 6.4. Energy Efficiency

In this regard, it is essential to measure and analyze the tradeoff between an application’s performance and familiarizing power allocation as a power-saving mode on cellular devices. Additionally, analyzing the effectiveness of beamforming algorithms across a large scale demands more attention. Harvesting energy from renewable resources can increase the ultra-dense CRANs’ performance from a perspective of energy efficiency. It is also imperative to enquire about efficient RRH switching-off schemes to minimize the consumption of energy using fewer traffic scenarios.

### 6.5. Network Scalability

The channel state information (CSI) has been always demanded improvement. Though the stochastic beamforming scheme has been discussed in the previous literature as a way to minimize CSI acquisition excess, it still requires a more effective algorithm for large-scale networks. Moreover, the uplink compression techniques can be improved to enhance the sum-rate capacity. Heuristic algorithms should also be developed for effective Infrastructure Deployment and Layout Planning (IDLP) on a large scale. Furthermore, heuristic algorithms for time efficiency demand more attention for minimizing the complex challenges of network scalability.

### 6.6. Mobility Management

Offering continuous and robust connectivity over various cellular technologies of communication is crucial for moving automobiles. In this regard, it is essential to examine the utility of operations and improved algorithm designs that have the least complexity and which depend on network operator or user-based necessities. Because the patterns of mobile call correlation develop extreme patterns of identical BS at the same time in a coverage area, designing mobility-aware adaptive techniques for effective optimization is an issue that will demand attention in future research.

### 6.7. Management of Services

It is essential to calculate network parameters such as traffic conditions and sparsity in network topology; therefore, the signaling design for the better performance of the CRAN system can be modified accordingly.

### 6.8. Network Virtualization

To improve end-to-end performance, it is necessary to investigate wireless network virtualization. Communication having one user in a virtual cell is not a suitable approach. This will result in interference when coming closer to other users. However, to maintain the benefits of minimized interference by multiuser cooperative transmission, it is essential to examine reliable virtualization techniques to avail multiuser cooperative transmissions. Evolving network slicing strategies can also be examined to facilitate 5G heterogeneous services containing low-latency and ultra-reliable communications, massive machine-type communications, and enhanced mobile broadband.

### 6.9. Appropriateness in Practical Situations

It is essential to deploy the proposed schemes in field tests and segregate them from the literature to examine their appropriateness in practical situations. Furthermore, ML techniques and aggregation tactics for online learning-based guidelines could be examined in genuine situations with unknown network parameters and differences across time. Therefore, most theoretical studies extracted from the literature need to be confirmed practically, which demands the development of experimental prototypes and future research in real-world measurement-based trials and analysis.

## 7. Conclusions

This review paper conducted an organized examination of resource allocation schemes and techniques that have been presented by different researchers. Our review also addresses the problems, policies or algorithms, and improvement of results. Based on several readings of studies presented in this research paper, we investigated those numerous methods that did not take into consideration several essential standards and assert that boosting the proficiency of the current methods is important. This finding on its own permits researchers to carry out further exploration in their upcoming research to enhance the field’s general competence in addressing resource allocation in 5G. 5G is a developing technology that would allocate substantial resources to enhancing QoS and system accomplishments. Additional work on allocating resources is desirable. Likewise, broad investigation on resource allocation methods that affect the green optimization of the base station would be admirable. The intent of this survey was to encourage additional practical study of resource allocation for 5G.

## Figures and Tables

**Table 1:** List of Keywords and Strings.

String	B1	B2
String 1 (S1)	Fifth Generation	Resource Allocation
String 2 (S2)	Fifth Generation	Resource Distribution
String 3 (S3)	Fifth Generation Network	Resource Reservation
String 4 (S4)	5G	
String 5 (S5)	5G Network	

**Table 2:** Data Sources.

Publisher	URL
MDPI	https://www.mdpi.com (accessed on: 1 July 2021)
Science Direct	https://www.sciencedirect.com (accessed on 15 June 2021)
Wiley Online Library	https://onlinelibrary.wiley.com (accessed on 20 June 2021)
Springer	https://link.springer.com (accessed on 25 May 2021)
Sage	https://journals.sagepub.com (accessed on 12 May 2021)
Google Scholar	https://scholar.google.com (accessed on 25 July 2021)
ACM	https://www.acm.org (accessed on 20 May 2021)
IEEE	https://ieeexplore.ieee.org (accessed on 28 June 2021)

**Table 3:** Inclusion and exclusion criteria.

Criteria	
Inclusion	⮚ The articles were published in well-reputed journals and conferences. ⮚ The article was peer-reviewed. ⮚ The article was written in English. ⮚ The study focused on resource allocation in 5G. ⮚ The article was published by the abovementioned publishers.
Exclusion	⮚ The articles were from keynote speeches, editorials, and white papers. ⮚ The articles were other than the English language. ⮚ The articles were not peer-reviewed. ⮚ The articles focused on issues other than resource allocation in 5G.

**Table 4:** Characteristics of selected resource allocation techniques in 5G.

Ref	Algorithm/Scheme/Strategy	Problem Addressed	Improvements/Achievements	Limitations/Weakness
[89]	Cooperative Online Learning Scheme	Extreme interference between the multi-tier users.	Maximizes spectral efficiency data rate by ensuring QoS.	Limited to tier and for downlink.
[90]	Game-theoretic approach	Cross-tier interference.	Improves spectral efficiency.	Limited to two tiers only.
			Energy efficiency.	Does not support uplink.
			Sum rate.	
[91]	Genetic Algorithm Particle Swarm Optimization-Power Allocation (GAPSO-PA)	The allocation of power in heterogeneous ultra-dense networks.	Reduces the system outage probability.	Solves non-linear optimization.
[92]	Estimation of Goodput based Resource Allocation (EGP-BASED-RA)	Enhance Goodput (GP): (a specific metric of performance).	The performance of the UFMC system was boosted.	Limited to a particular packet format.
[93]	The social-aware resource allocation scheme	D2D multicast grouping;	Fairness.	Working on limited parameters.
		Ineffective D2D links.	Throughput.	
			Has substantial benefits over other algorithms.	
[94]	PGU-ADP algorithm	Dynamic virtual RA problem.	Drastically minimizes the outage probability.	Considers specific slice rate.
		Expansion of the total user rate.	Enhances user data rate	Slice state.
				Downlink only.
[95]	Efficient Resource Allocation Algorithm	Enhance system capacity and maximum computational complexity.	Improves system capacity.	Power allocation is done on the sub-carriers of the fixed group.
			Minimizes complexity performance.	Limited parameters.
[96]	GBD Based Resource Allocation Algorithm	Enhances allocating algorithm’s efficiency.	Total throughput achieved 19.17%.	Parameters are not suitable in all circumstances.
			Average computational time 51.5%.	
			GBD with no relaxation by30.1%.	
[97]	Multitier H-CRAN Architecture	Lacking intelligence perspective using existing C-RAN methods.	Manages spectral resources efficiently.	Necessary to improve bits of intelligence.
			Enhances control.	
			End-to-end optimization.	
			Ensures QoS by 15%.	
[98]	Bankruptcy game-based algorithm	Resource allocation and inaccessibility of wireless slices.	Enhances resource utilization.	Focused on the cloud—RAN.
			Ensures the fairness of allocation.	Limited to specific parameters and slices.
[99]	BVRA-SCP Scheme	Enhancing service demands like low latency, enormous connection, and maximum data rate.	Beneficial resource utilization.	Limited to dynamic IoT-specific metrics.
			Low computational complexity.	
			Supports dynamic IoT. slicing architecture.	
			Improves efficiency and flexibility.	
[100]	VNF-RACAG Scheme	Settlement of virtualized network functions (VNF).	The gain in end-to-end delay.	Limited parameters.
[101]	Hybrid DF-AF scheme	Promising to incorporate various wireless networks to deliver higher data rates.	Attains the concave envelope of the maximum between AF rate and DF rate.	Limited parameters are considered.
			Substantial gains for RFDRC.	
[102]	Cooperative resource allocation and scheduling approach	Scheduling and resource allocation problems.	Decreases transmission collision probability.	Only for URLLC traffic.
			Enhances the reliability of upcoming 5G.	Considers limited parameters.
			Enhances vehicle-to-everything (ev2x) communications.	
[103]	SWIPT framework	Low energy efficiency and high latency.	Maximizes energy efficiency.	Limited to downlink.
			Effective capacity.	Considers limited metrics.
[104]	The device-centric resource allocation scheme	Declining of network throughput and raises delay in resource allocation.	Reduces load at the BS up to 35%.	Improvement is required in intelligent resource allocation.
			Better performance.	Power efficiency was neglected.
[105]	Distributed Resource Allocation Algorithm	Resource allocation and interference management in 5G networks.	Efficient higher data rate results.	Limited to uplink only.
				Limited parameters were used.
[106]	Unified cross-layer framework	Physical layer modulation format and waveform, resource allocation, and downlink scheduling.	Enhances spectral efficiency using FBMC/OQAM.	Limited to specific parameters and frequency.
[107]	Dynamic joint resource allocation and relay selection scheme	Relay selection and downlink resource allocation.	Low computational complexity.	QoS neglected.
				Limited metrics are considered.
[108]	Low-Complexity Subgrouping scheme	Radio resource management of multicast transmissions.	Improves the Aggregate Data Rate (ADR).	Focused on data rate only.
			Ensures performance up to 9%.	QoS neglected.
				Limited parameters.
[109]	Joint Edge and Central Resource Slicer (JECRS) framework	Requires distinct resources from the lower tier and upper tier.	Satisfies latency and resource requirements.	Needs to support the NFVO.
			Guarantees communication and computing.	
[110]	TCA algorithm	MTC devices are battery restricted and cannot afford much power consumption needed for spectrum usage.	Less complex.	N/A
			Achieves better performance.	
[111]	IHM-VD algorithm	Power allocation and channel allocation issue.	Outperforms energy efficiency.	Focuses on specific parameters and particular domain.
			QoS requirements.	
[112]	Centralized approximated online learning resource allocation scheme	The inter-tier interference among macro-BS and RRHS; and energy efficiency.	Ensures interference mitigation.	Limited to inter-tier interference mitigation.
			Maximizes energy efficiency.	Limited to specific parameters.
			Maintains QoS requirements for all users.	
[113]	Spectrum resource and power allocation scheme	Emphasize on a fair distribution of resources in one cell.	Boosts system performance.	Limited to user interference in a single cell.
				Not suitable for multiple cell interference.
				QoS neglected.
[114]	Tri-stage fairness scheme	Resource allocation problem in UDN having caching and self-backhaul.	Improved flexible access and backhaul link resource allocation.	Particularly uses caching.
				Limited parameters are used.
				QoS is neglected.
[115]	Fronthaul-aware software-defined resource allocation mechanism	Overhead generated using a capacity-limited shared fronthaul.	Throughput enhancements.	Limited to in-band fronthaul.
			Delay reductions.	Limited parameters are used.
[116]	Heterogeneous statistical	Heterogeneity issues.	Efficient QoS across MIMO-OFDMA based CRNS.	Domain-specific.
	The QoS-driven resource allocation scheme			Limited parameters are used.
				Limited to effective capacity.
[117]	Nondominated sorting genetic algorithm II (NSGA-II)	Unable to get optimal results concurrently.	Performance.	Limited to ultra-dense network.
			Analyzes computational and convergence complexity.	Limited to downlink.
[118]	Joint access and fronthaul radio resource allocation	Downlink energy efficiency (EE) and millimeter-wave (MMW) links in access and fronthaul.	The system sum rate is enhanced up to 50%.	Limited parameters.
			Using PD-NOMA and comp the sum rate was enhanced up to 40%.	RAN-based only.
				Limited to downlink.
[119]	Double-sided auction-based distributed resource allocation (DSADRA) method	Intercell and inter tier interference.	User association satisfaction.	QoS not considered.
			Maximum output.	Limited for small cell only.
[120]	Joint power and reduced spectral leakage-based resource allocation	Interference from D2D pairs.	Reduces spectral leakage to nearby RBS.	QoS neglected.
			Ensures maximize signal-to-interference-and-noise ratio (SINR).	Limited parameters.
			Enhances overall throughput	
[121]	Branch-and-bound scheme	Latency-optimal virtual resource allocation.	Enhances serviceability.	Limited to backhaul.
			Network load balance.	Limited parameters.
				Neglects energy efficiency.
[122]	The learning-based resource allocation scheme	To achieve high system capacity better performance in terms of effective system throughput.	More efficient in terms of system performance.	Limited to user’s position information.
[123]	Resource allocation method with minimum interference for two-hop D2D communications	Interference which reduces network throughput.	Enhances interference and throughput.	Limited parameters.
				Priority-based allocation block.
[124]	Multiband cooperative spectrum sensing and resource allocation framework	Energy consumption for spectrum sensing.	Satisfies the QoS requirement.	Channel fading changes over time.
				Mobile IoT nodes do not consider.
[125]	Channel-time allocation PSO Scheme	To acquire gigabit-per-second throughput and low delay for achieving and maintaining the QoS.	Encounters the growing requirements of applications.	Especially for multimedia traffic.
			Converged and high-capacity networks such as 5G.	Certain metrics are considered.
[126]	Heterogeneous (high density)/hierarchical (low density) virtualized software-defined cloud RAN (HVSD-CRAN).	Density of users.	Encounters variety of tradeoffs in resource management objectives such as cost, power, delay, and throughput.	Limited resource allocation in dense users.
				Certain parameters are used.
[127]	Mini slot-based slicing allocation problem (MISA-P) model	The probability of forming 5G slices.	Spectral efficiency and feasibility.	Limited parameter.
				Support single slot-based model.
				Limited for EMBB and URLLC traffic.
[128]	A joint resource allocation and modulation and coding schemes	Requirement of extremely low latency and ultra-reliable communication.	Achieves low error rates.	Only for URLLC traffic.
			Minimizes resource consumption.	Reserves resources for the first transmission.
[129]	QoS/QoE-aware relay allocation algorithm	Neglects temporal requirements for optimum performances.	Better performance for mean time to failure (MTTF).	Working based on different priorities.
			Average peak signal-to-noise ratio (PSNR).	Considers specific parameters.
			Average energy consumption.	
[130]	The learning-based resource allocation scheme	Interference coordination complexity and significant channel state information (CSI) acquisition overhead.	Better effective system performance.	Accuracy varies as per user positions.
				Neglects throughput and QoS.
[131]	Device-to-device multicast (D2MD) scheme	Improving spectrum and energy efficiency and enabling traffic offloading from BSs to device.	Throughput enhanced.	Lack of attention to mobile users.
				Neglects selection of sharing mode and content caching in D2MD.
[132]	Constrained deferred acceptance (DA) algorithm and a coalition formation algorithm	The interference management among D2D and current users.	Enhances performance.	Limited coverage area.
			Throughput, fairness, and admitted users.	Neglects reliability and security.
[86]	Novel resource allocation schemes (hybrid resource management)	Energy efficiency and consumption.	QoS threshold and power budget are ensured.	Lack of attention to delay and overhead.
[133]	Orthogonal multiple access (OMA) and relay-assisted transmission schemes.	Jointly optimize the block length and power allocation for reducing error probability.	Improves performance.	Emphasis on short packet transmission only.
				QoS is neglected
[134]	Joint user association and Power Control algorithm	Optimizing power control and user association schemes.	Achieves higher energy efficiency performance.	Lack of attention to fairness and channel state information.
[135]	Successive convex approximation (SCA) based alternate search method (ASM)	Raise the total sum rate of users.	Enhances the performance of the system.	Lack of attention to fairness.
			Ensures the potential of SCMA.	Limited parameters are used.
[136]	An online learning algorithm for resource allocation	Inter-tier interference among RRHS and macro-BSs, and energy efficiency.	Enhances the energy efficiency.	Priority-based allocation of the resource block.
			Maintains users’ QoS.	Limited parameters are focused.
[137]	Joint resource block (RB) and power allocation scheme	Enhance fairness in data rate among end-users.	Low complexity.	Limited to femtocell only.
			Higher spectral efficiency.	Interference inside femtocell not considered.
[138]	Hybrid multi-carrier non-orthogonal multiple access (MC-NOMA)	Achieve the SE-EE tradeoff having minimum rate requirement of each user.	Outperforms both NOMA and OMA.	Decreases performance while adding more users.
			Enhances the tradeoff between system efficiency and user fairness.	Complexity.
[139]	Stackelberg game model	High inter-cell interference (ICI) and less energy efficiency.	Feasible and promising.	Focuses on limited parameters.
				Neglects intra-cell interference.
[140]	Virtual code resource allocation (VCRA) approach	Reducing the collision probability.	Reduces the collision probability.	Improves the code.
			Enhances efficiency.	Access to devices is according to priority.
[141]	Deep reinforcement learning -unicast-multicast resource allocation framework (DRL-UMRAF)	High-quality services and achieving green energy savings of base stations.	Improves energy efficiency.	Limited services framework.
			QoS requirements.	Limited to the number of cells and layers.
[142]	Deep reinforcement learning-based intelligent Up/Downlink resource allocation	The high dynamic network traffic and unpredicted link-state change.	Performance improvement.	Lack of attention to overhead.
			Packet loss rate and network throughput.	QoS neglected.
[143]	Joint computation offloading and resource allocation scheme	Complete network information and wireless channel state.	Outperforms energy consumption.	Limited to a specific parameter.
				QoS is neglected.
[144]	Deep neural network-Multi objective Sine Cosine algorithm (DNN-MOSCA)	Achieving better accuracy and reliability.	Better performance.	Spectral efficiency was neglected.
			Improves fairness, throughput, and energy efficiency.	
[145]	The improved resource allocation algorithm	Improving QoS requirements in MTC.	Expressly improves the outage and success probability.	Prioritizes access for MTC devices.
				Limited parameters are considered.
[146]	Resource Allocation Algorithm	The interference to 5G cellular users (CUs) related to QoS.	Improves the cellular users’ channel capacity.	Limited parameters are considered.
			Guaranteeing QoS of the CUs.	Only for uplink.
[147]	Genetic algorithm- intelligent Latency-Aware Dynamic Resource Allocation Scheme (GI-LARE)	Efficient radio resource management.	GI-LARE outperforms these other schemes.	Divides traffic into 2 categories.
				Downlinks only.
				Specific parameters were used.
[148]	A Low-complexity centralized packet scheduling algorithm	Downlink centralized multi-cell scheduling.	Improves URLLC latency.	Neglects inter-cell interference.
			Achieves gains of 99% and 90% URLLC latency.	Considers only URLLC traffic.
[149]	Smart queue management method	QoS of end-to-end real-time traffic.	Confirms better end-to-end communication QoS of the real-time traffic.	Not for all IoT critical services.
			The average end-to-end communication delay was reduced.	Neglects other relevant parameters.
[150]	Proposed Optimal Resource Allocation Algorithm	The optimization problem in mixed-integer nonlinear programming (MINLP).	Improves throughput.	Wi-Fi or LTE only.
			Guarantees QoS of Wi-Fi user equipment.	Limited parameters are used.
				Good in one scenario only.
[151]	A novel packet delivery mechanism	Issues related to using CoMP for URLLC in C-RAN architecture.	Resource utilization.	Limited for URRLC traffic.
			UE satisfaction.	Lack of attention to overhead.
[152]	Distributed joint optimization algorithm for user association and power control	Improve total energy efficiency and reduce the inter-cell and intra-cell interference.	Effective and robust dynamic communication environment.	Limited to two-tier.
				Lack of attention to overhead.
[153]	Pollaczek–Khinchine formula based quadratic optimization (PFQO)	Inaccurate transmission recovery delay of URLLC multi-user services.	Bandwidth saving.	Lack of attention to retransmission timing.
			Packet length distributions.	Specific parameters.
[154]	An outer approximation algorithm (OAA)	Multiple interferences, imbalanced user traffic load.	Mitigating interference.	Lack of attention to QoS.
			Traffic offloading to address traffic imbalances.	Latency.
			Sum-rate maximization.	
[155]	Joint Power and Subcarrier Allocation	URLLC reliability and network spectral efficiency.	Improves the spectral efficiency.	Limited to a single cell.
			URLLC reliability.	Not allocated slices in multiple cells.
				Neglects overhead.
[156]	Weighted Majority Cooperative Game Theory Based Clustering	Increase interference, improper utilization of resources.	Power consumption decreases up to 30%.	Fairness is not considered.
			SINR and spectral efficiency are increasedup to 40% and 45%, respectively.	Prioritizes small cells based on weight.
[157]	Bee-Ant-CRAN scheme	Design a logical joint mapping among RRHS and User Equipment (UE) and RRHS and BBUS too.	Improves the spectral efficiency as well as the throughput.	Neglects the effect of virtual BS.
				Lack of attention to energy efficiency.
[158]	Noncooperative game theory-based user-centric resource optimization scheme	Enhance the coverage probability and sum rate.	Improves the sum rate.	Limited to single macro cell scenario.
			Outage probability.	Neglects energy efficiency.

**Table 5:** Metrics used in 5G Resource Allocation.

Metrics	References
Response Time	[119]
End-To-End Delay	[100,149]
Delay	[94,103,104,106,115,116,124,125,126,148,153]
Throughput	[89,93,96,97,98,104,106,107,108,110,115,120,121,122,123,124,125,126,131,137,142,144,150,158]
Packet Loss	[142,147]
Latency	[102,103,109,115,121,128,133,147,148,151]
Overhead	[95,114,122,130]
Jitter	[96]
Availability	[93,121]
Spectral Efficiency	[89,90,92,95,97,105,112,113,117,127,136,137,138,155,156,158]
Fairness	[89,93,96,98,106,107,113,114,132,137,138,144]
Outage Ratio	[89,94,145]
Sum Rate	[90,94,101,103,113,118,135,146,154,158]
Energy Efficiency	[86,90,97,103,110,111,112,114,118,124,134,136,138,139,140,141,142,143,144,152]
System Performance	[91,95,99,100,101,105,108,113,117,122,132]
Low Complexity	[91,95,96,98,99,107,108,110,114,117,130,132]
Power Allocation	[86,91,92,93,94,95,97,110,111,113,118,119,120,132,134,139,152]
Reliability	[102,124,128,133,155]
Time Required for RA	[104]
Scalability	[108]
Interference	[100,112,113,119,120,132,146,152,154,156]
Power Consumption	[126,156]
Feasibility	[128]
Energy Consumption	[129,143]

**Table 6:** Uplink Downlink with Domains.

Domain	References
Fronthaul	[86,89,90,93,94,95,99,101,103,104,105,106,107,109,110,111,113,114,115,117,118,120,123,124,129,133,134,137,138,139,140,142,144,145,146,147,149,150,152,153,154,155,156,158]
C-RAN	[96,97,109,111,112,118,119,122,126,130,135,143,148,151,157]
H-CRAN	[97,132,136,143]
Backhaul	[99,110,111,114,133,147]
Uplink	[96,97,99,104,105,109,110,111,113,119,120,123,124,129,140,142,143,145,146,149,150,153,154,156,157,158]
Downlink	[86,89,90,94,95,96,97,99,101,103,104,106,107,109,111,112,114,115,117,118,120,122,126,129,130,132,133,134,135,136,137,138,139,142,143,144,147,148,149,151,152,153,154,155,157]
